# Secondary Analysis to Evaluate Performance Expression Stability of Alternative Complex–Contrast Training Set Strategies

**DOI:** 10.3390/mps9020062

**Published:** 2026-04-03

**Authors:** Liam J. Houlton, Jeremy A. Moody, Theodoros M. Bampouras, Joseph I. Esformes

**Affiliations:** 1 School of Sport, Cardiff Metropolitan University, Cardiff CF23 6XD, UK; st20090559@outlook.cardiffmet.ac.uk (L.J.H.); jesformes@cardiffmet.ac.uk (J.I.E.); 2 Faculty of Sport, Health and Fitness, City College Plymouth, Plymouth PL1 5QG, UK; 3 School of Physical Education and Sports, Nişantaşı University, Istanbul 34398, Turkey; 4 School of Sport and Exercise Sciences, Liverpool John Moores University, Liverpool L3 3AF, UK; t.bampouras@ljmu.ac.uk

**Keywords:** consistency, impulse, power, resistance training, strength

## Abstract

This study aimed to evaluate the performance expression stability (PES) of sixteen alternative complex–contrast training (CCT) set strategies. Three separate cross-sectional studies (*n* = 14–17) evaluated the effects of different intra-contrast rest periods (ICRP; ≤300 s) and rest redistribution (RR) strategies (≤60 s) within CCT sets on the application of vertical jump propulsive force were examined using dual force platforms. To establish PES for propulsive force–time variables, repetitions one and two of the baseline set were analyzed using within-participant (coefficient of variation, CV; standard error of measurement; smallest worthwhile change; relative mean bias) and between-participant (intra-class correlation coefficient, ICC_3,1_; Pearson’s correlation coefficient, *r*) stability metrics. Results showed that all CCT set strategies facilitate stable performance expression between participants and facilitated the detection of practically meaningful changes for propulsive impulse, peak force, mean force, and propulsion time (ICC_3,1_ = 0.64–0.99, *r* = 0.80–0.99, CV = 1.12–9.98%), while rate of force development metrics demonstrated less consistent between- and within-participant stability (ICC_3,1_ = 0.55–0.97, *r* = 0.46–0.96, CV = 7.52–27.66%). The findings indicate that alternative CCT set strategies facilitate the stable expression of propulsive force–time performance in vertical jumps, although individualized prescriptions are essential for optimizing rate of force development outcomes. Performance expression stability insights provide a practical tool for balancing the effectiveness and potential for performance enhancement of vertical jump propulsion across alternative CCT set strategies. Practitioners may use these results to improve the prescription and monitoring of CCT-based strength and power mesocycles.

## 1. Introduction

Complex–contrast training (CCT) is an advanced training method that alternates a biomechanically similar, high-load conditioning activity and a high-velocity explosive activity to acutely enhance neuromuscular performance through post-activation performance enhancement (PAPE) [1,2]. It is theorized that the conditioning activity enhances neuromuscular performance of the subsequent explosive activity by increasing the rate and synchrony of high-threshold motor unit recruitment, intramuscular water content, muscle temperature, and changes in pennation angle [3,4]. Alongside neuromuscular enhancement, high-load conditioning activities simultaneously elicit high levels of acute fatigue and movement pattern interference, whereby the slow conditioning activity contraction speed is maintained during the explosive activity [1,3,4]. However, it is understood that fatigue dissipates faster than excitation [4]. Thus, PAPE is the net product of excitation and fatigue, whereby adequate rest between conditioning and explosive activities will manifest as enhanced explosive activity performance. This is typically observed through augmented rate of force development (RFD) and propulsive impulse (J_PROP_) [4,5]. Previous meta-analyses have demonstrated that the rest period, known as the intra-contrast rest period (ICRP), required for PAPE to manifest is 5–12 min [1,6]. The duration of the ICRP is highly individualized and depends on numerous factors, such as strength, experience and muscle fiber type [6]. However, whilst useful in clinical environments, 5–12 min is typically not viable in many athletic training scenarios, where training time is often limited. In these scenarios, rest periods ≥ 5 min may be inappropriate, suggesting that CCT may be impractical as a training method.

To address practicality, alternative set strategies may be combined with CCT prescriptions to limit conditioning activity-induced fatigue accumulation and movement pattern interference, while maintaining the prescribed conditioning activity volume load. This may facilitate the earlier manifestation of PAPE. Rest redistribution (RR) is an alternative set strategy that splits traditional resistance training sets into smaller subsets of repetitions or individual repetitions by reorganizing prescribed inter-set rest periods [7,8]. The current literature repeatedly demonstrates the effectiveness of RR strategies for maintaining barbell mean velocity and power for more repetitions than traditional sets [9,10,11,12,13,14], by facilitating partial metabolic recovery through phosphocreatine and adenosine triphosphate replenishment [8] and increasing intramuscular pH through partial lactate buffering [15,16]. Theoretically, facilitating short rest periods between conditioning activity repetitions within CCT prescriptions may limit fatigue accumulation and interference with movement patterns, thereby allowing earlier detection of potentiated explosive activity performance than CCT research currently suggests.

Based on this rationale, we previously conducted three independent cross-sectional studies [17,18,19]. These studies aimed to assess the effect of 0–300 s ICRPs on subsequent countermovement jump (CMJ) and squat jump (SJ) propulsive force after performing a conditioning activity consisting of a three-repetition maximum (3RM) back squat. Rest periods up to 300 s were assessed, as this was considered the upper threshold for practical application in many athletic training scenarios where training time is limited. Houlton et al. [17] assessed the effect of traditional conditioning activities and ICRPs, meaning the conditioning activity was continuous and the ICRP was not redistributed. Houlton et al. [18] assessed the effect of 30 s RR, in which 30 s of the ICRP was redistributed into 15 s rest periods between conditioning activity repetitions. Houlton et al. [19] assessed the effect of 60 s RR, where 60 s of the ICRP was redistributed as 30 s rest periods between conditioning activity repetitions. Based on these experimental designs, the total contrast rest periods (TCRPs), defined as the sum of intra- conditioning activity rest and ICRPs, were the same across all three studies. The results of these studies demonstrated that neither TCRP duration nor RR strategies affected J_PROP_ and therefore CMJ or SJ height [20]. Houlton et al. [19] demonstrated peak and average RFD (RFD_PEAK_ and RFD_AVE_, respectively) enhancement and reduced propulsion time (t_PROP_) after 180, 240 and 300 s TCRPs, suggesting that redistributing larger portions of longer rest periods may enhance explosive performance via the propulsion strategy. However, the reduction in proximity to fatigue may have limited high-threshold motor unit recruitment [21,22] and, subsequently, the total force applied during vertical jumps. Furthermore, force–time measures like RFD are generally considered noisy metrics [23,24,25], which may compound with observed individuality of PAPE, reducing the likelihood of observing effects. This makes it difficult to ascertain the performance expression stability (PES) of these CCT prescriptions in athletic populations.

The highly variable and individualized nature of acute PAPE responses indicates that traditional pairwise or means-based analyses may be insufficient for determining whether a given CCT set strategy facilitates stable performance expression within or between participants. In CCT contexts, athletes with similar strength and training status may display different magnitudes of performance enhancement over different time courses [26,27]. Consequently, alternative CCT set strategies may not lack practical utility; rather, they may result in heterogenous but repeatable potentiation effects (i.e., within-participant performance enhancement is similar across multiple exposures to similar stimuli) that require assessment of their PES. Performance expression stability is operationally defined here as the ability of an examined intervention to induce a consistent response and, thus, be used as a training stimulus in multiple training sets within a session.

The PES of CCT set strategies may be determined by quantifying both measurement consistency and the detectability of meaningful change. Within-participant analysis of stability using coefficient of variation (CV) and standard error of measurement (SEM) quantifies measurement noise and within-participant variability [28]. These indices may be used to ascertain how consistently specific CCT set strategies facilitate replication of within-participant performance outcomes. When combined with the smallest worthwhile change (SWC), these indices indicate whether a CCT set strategy facilitates true, practically meaningful changes in force–time metrics that can be distinguished from random variation [29,30]. However, within-participant analyses alone do not inform practitioners about PES at the group level, which is critical for programming and monitoring in applied settings where training systems are typically designed with team-based constraints. This is particularly apparent when assessing conditions that typically elicit heterogeneous performance responses, like CCT strategies [31].

Thus, alongside within-participant analyses, between-participant indices like the intraclass correlation coefficient (ICC) and Pearson’s correlation coefficient (*r*) may be used to evaluate whether CCT set strategies preserve stable performance expression between athletes across repeated exposures within a training session [32]. High between-participant stability may suggest that participant differences in the ability to express within-session potentiation represent stable characteristics rather than random fluctuations, supporting the use of CCT prescriptions to differentiate between responders, non-responders, negative responders, and, subsequently, athletes requiring individualized manipulation of training variables. In the context of CCT, where PAPE is strongly influenced by neuromuscular and fatigue-recovery profiles, integrating PES analysis allows practitioners to judge not only whether a set strategy can effectively facilitate performance enhancement of the chosen propulsion metrics but whether it does so consistently enough to inform training prescription and monitoring at the individual and group level.

Accordingly, there is a need to move beyond evaluating whether CCT set strategies are effective through group-based means and pairwise comparisons, by integrating the assessment of within- and between-participant PES analysis alongside inferential statistics to account for the individualized and noisy nature of CCT potentiation. By quantifying within-session stability, measurement error and meaningful changes, PES analysis can clarify which CCT set strategies are suitable for enhancing or monitoring key metrics and those that result in inconsistent, highly variable changes. Thus, practitioners can balance the PES with pairwise effects to establish the performance potential of alternative CCT set strategies. Therefore, this study aimed to examine the within-session PES of 16 different CCT set strategies for eliciting potential performance changes in CMJ and SJ propulsive force–time metrics. It was hypothesized that incorporating RR within CCT sets would enhance the within- and between-participant PES of CCT strategies by inhibiting fatigue and movement pattern interference thereby improving the potential for athletes to express potentiation through acute vertical jump propulsion.

## 2. Materials and Methods

### 2.1. Participants

Sample demographic information for Houlton et al. [17,18,19] is presented in Table 1. All participants were male, recreational athletes. Aligned with definitions from sports science literature [33,34], for these studies, a recreational athlete was defined as a person who trained for general strength, two to four times a week, with no focused strength and conditioning program aligned to sports performance. Participants were required to have had no musculoskeletal injuries that could impede data collection (e.g., lower-limb or torso injuries) for at least 3 months before data collection. They were required to have at least 1 year of resistance training experience, demonstrate technical competency in the back squat, CMJ, and SJ, in line with the technical models presented in Brewer and Favre [35], Acero et al. [36], and Arabatzi et al. [37], respectively, and demonstrate back squat relative strength > 1.50. Relative strength level was set at 1.50–2.00, as this is representative of average to good strength levels sought by coaches in many athletic training scenarios [38,39] and is similar to the inclusion criteria from similar CCT-based research [40,41,42,43]. Technical competency was confirmed during an initial familiarization session. Back squat 3RM loads were self-reported for inclusion. The back squat 3RM was then confirmed through a formal maximal strength assessment following the guidelines set out in Sheppard and Triplett [44]. Familiarization and maximal strength assessments were separated by at least 48 h. If participants did not achieve a back squat 3RM relative strength > 1.50 during the formal assessment, they were removed from the study. With awareness of potential hyper-familiarization effects [45,46], the research team made every effort to recruit unique samples for each cross-sectional study. However, two participants who were recruited took part in all three cross-sectional studies, four participants took part in Houlton et al. [17,18], and two participants took part in Houlton et al. [18,19].

Upon recruitment, participants were briefed on the data collection requirements, provided with a participation information sheet, and informed of their right to withdraw from the study at any time. Participants then completed a health questionnaire and provided informed consent. The Institutional Review Board at The School of Sport and Health Sciences, Cardiff Metropolitan University, provided ethical approval for all three studies (Houlton et al. [17], protocol code: PGR-5893, approved on 3 August 2022; Houlton et al. [18], protocol code: PGR-7475, approved on 6 April 2023; Houlton et al. [19], protocol code: PGR-7607, approved on 18 May 2023).

### 2.2. Cross-Sectional Study Experimental Procedures

**Figure 1:**
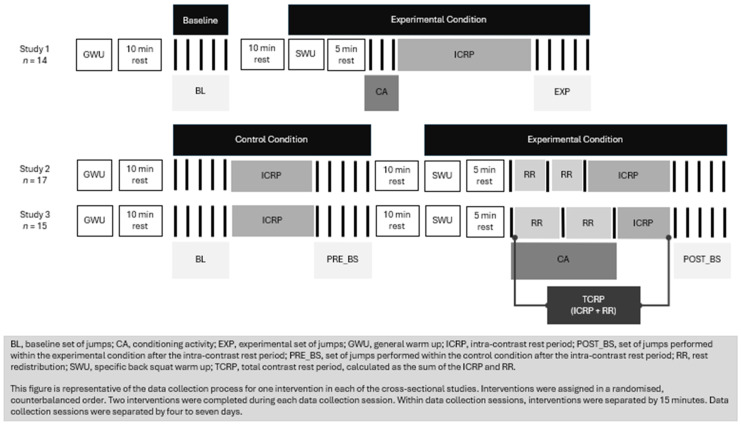
Cross-sectional study design. Study 1, Study 2 and Study 3 refer to Houlton et al. [17], Houlton et al. [18] and Houlton et al. [19], respectively.

Prior to this secondary analysis, three separate cross-sectional studies were conducted [17,18,19]. Each study assessed the effect of a CCT set strategy on vertical jump propulsive force. All studies employed cross-sectional, counterbalanced, repeated measures designs. Briefly, Houlton et al. [17] assessed the effect of a conditioning activity followed by an ICRP of up to 300 s on vertical jump propulsive force application within a CCT set. In Houlton et al. [17], participants performed a baseline set of jumps (BASELINE), followed by an experimental condition. The experimental condition consisted of the conditioning activity (3RM back squat), followed by an ICRP, followed by an experimental set of jumps (EXP). The 3RM back squat was performed traditionally, i.e., continuously, with no rest between repetitions. Houlton et al. [18] and Houlton et al. [19] aimed to assess the effect of an RR strategy within a CCT set on the application of vertical jump propulsive force. In both studies, participants performed a control condition followed by an experimental condition for each set of independent variables. The control condition consisted of a baseline set of jumps (BASELINE), the prescribed ICRP, and a second set of jumps (PRE_BS). Then, participants performed an experimental condition. This consisted of the conditioning activity (3RM back squat), followed by the same ICRP assigned to the control condition, followed by a third set of jumps (POST_BS). In Houlton et al. [18] and Houlton et al. [19], the 3RM back squat repetitions were separated by 15 s and 30 s rest, respectively. The sum of the rest between conditioning activity repetitions and the ICRP was labeled as the TCRP. The TCRPs in Houlton et al. [18] and Houlton et al. [19] were equal to the ICRPs assessed in Houlton et al. [17]. The data collection processes for all three studies are summarized in Figure 1, and independent variables (TCRP, including the organization of the rest within experimental conditions, and vertical jump variant) are presented in Table 2. For full details of the experimental designs and data collection processes, the reader is directed to the respective papers [17,18,19].

In all three studies, vertical jump ground reaction force data were collected using dual force platforms (PS-2141, Pasco, Roseville, CA, USA) with a sampling rate of 1000 Hz and recorded using commercial software (Pasco Capstone 2.0, Pasco, Roseville, CA, USA). Pasco Capstone software does not facilitate signal filtering; as such, CMJ and SJ raw data were downloaded as CSV files and transferred to respective custom-made Microsoft Excel (Microsoft, Redmond, Washington, USA) templates to extract the dependent variables (Table 3). All data were processed identically to enable between-study analysis. For information regarding definitions of CMJ and SJ propulsive phases, the reader is directed to Linthorne [47], McMahon et al. [48] and Perez-Castilla et al. [25]. For information on dependent variable definitions and calculations, see Boullosa et al. [49] and Perez-Castilla et al. [25] for CMJ variables and Hansen et al. [50], McLellan et al. [24] and Torres-Laett et al. [51] for SJ variables.

### 2.3. Data Analysis

To assess the PES of vertical jump propulsion metrics within multiple alternative CCT set strategies, it was deemed appropriate to assess the stability of BASELINE repetitions. Assessment of PES requires no intervention between assessed repetitions, as biological changes (e.g., excitation or fatigue) will affect subsequent performance. As such, stability metrics were calculated from BASELINE data to ascertain raw stability and facilitate subsequent use in assessing performance potential by combining PES with inferential pairwise comparisons

The methodologies used for data collection in the three cross-sectional studies were prospectively harmonized in line with guidelines set out in Cheng et al. [52]. This facilitated robust secondary analysis without taking steps to normalize or transform data. As experimental designs differed slightly across studies, all data analysis was completed for repetitions one and two of the BASELINE set of vertical jumps for each set configuration. The residuals (differences between pairs) and the respective means between pairs for each set configuration and dependent variable were correlated to assess heteroscedasticity [53]. All correlations were non-significant, facilitating analysis of the raw values. As *n* ≤ 17 for all set configurations, normality was confirmed for all set configurations and dependent variables using Shapiro–Wilk’s test and through visual inspection of Q-Q plots. The strategy of using visual inspection and statistical testing for normality is frequently recommended [54]. Q-Q plots were examined by two members of the research team (L.J.H. and T.M.B.), supplemented by the results of the Shapiro–Wilk’s test, which is recommended for smaller sample sizes (e.g., *n* < 50) [55]. To determine within- and between-participant PES of alternative CCT set strategies for facilitating vertical jump propulsion metrics, within- and between-participant stability metrics were calculated for each dependent variable, following the recommendations of Atkinson and Nevill [31].

Within-participant PES indices were calculated to determine within-participant variance and the likelihood of detecting practically meaningful changes in performance using the CV with 95% confidence intervals (95% CI), relative mean bias (RMB) with 95% limits of agreement (95% LoA), SEM with 95% CI, and SWC with 95% CI. The CV was used to assess individual variance for each CCT set structure. Individual CVs were calculated for each participant by dividing the standard deviation by the mean and converted to a percentage and the overall CV was calculated as the mean of individual CVs [31]. In line with relevant literature focused on vertical jumping, a CV < 10% was considered acceptable [50,56,57]. The SEM was used to estimate the variation of values around individual true scores, representing the precision with which each propulsion metric can be manipulated using each CCT set strategy. It was calculated from the square root of the mean square error term of a repeated-measures ANOVA [28,58,59]. Relative mean bias with 95% LoA was calculated for each CCT set strategy to establish a practical boundary for the expected change in individual performance and the interval for expected variation in differences between paired measurements. Relative mean bias was calculated as the individual mean difference between paired measurements divided by the mean of the paired measurements [60]. The SWC with 95% CI was used to determine the minimal real change in performance for each CCT set strategy. Smallest worthwhile change values were calculated by multiplying the threshold for a small effect by the SD of the differences [61]. As the samples were considered recreational, SWC was calculated as 0.35 × SD, based on recommended adjustments to effect-size thresholds [62].

Between-participant PES indices were calculated to determine the sample PES of each CCT set strategy using intraclass correlation coefficients (ICC_3,1_) and Pearson’s correlation coefficient (*r*) with 95% CI. The two-way mixed-effects model (ICC_3,1_) was chosen to facilitate interpretation of results for random participants (i.e., outside the samples) and for single trials, rather than for the mean of multiple trials (i.e., aligned with the repeated exposure to CCT prescriptions in training scenarios) [63]. Intraclass correlation coefficients were used to estimate systematic biases of repeated scores for each CCT set strategy [64]. Coefficients in the ranges of 0.00–0.10, 0.10–0.30, 0.30–0.50, 0.50–0.70, 0.70–0.90, and 0.90–1.00 were classed as trivial, small, moderate, large, very large, and nearly perfect ICCs, respectively [65]. Pearson’s correlation coefficient was used to assess the PES between participants for each CCT set strategy. A high correlation between time points was observed when r > 0.80 [53,66].

The ICC_3,1_ values for each CCT set strategy were calculated independently. However, as a small number of participants appeared in more than one study, non-independence was introduced, which could potentially affect the results. Additional analysis was conducted to assess their impact by recalculating ICC_3,1_ values with shared participants removed from the analysis. No difference in ICC_3,1_ values between analyses was observed.

Unless otherwise stated, all data are presented as mean ± SD. Significance was set at *p* < 0.05. All statistical tests were performed in Microsoft Excel and JASP (version 0.19.1.0; JASP Team, Amsterdam, The Netherlands).

## 3. Results

### 3.1. Countermovement Jump

Descriptive statistics for the first and second jumps of the BASELINE set of CMJs across all CCT set strategies, along with within- and between-participant PES metrics for each dependent variable, are provided in Table 4. Correlation coefficients indicate that, across all CCT set strategies, ranked performance is preserved for J_PROP_, PF, MF, and t_PROP_ (*r* = 0.80–0.99). However, not all CCT set strategies facilitated the preservation of ranked performance for RFD_PEAK_ (*r* = 0.46–0.94), RFD_AVE_ (*r* = 0.61–0.96), and RFD_INDEX_ (*r* = 0.60–0.93). All CCT set strategies demonstrated moderate to nearly perfect ICCs for all dependent variables (ICC_3,1_ = 0.55–0.99).

Regarding within-participant PES, all CCT set strategies demonstrated acceptable CV values for J_PROP_, PF, MF and t_PROP_ (CV = 1.12–5.03%), suggesting acceptable within-participant PES. Unacceptable CV values were observed under nearly all CCT set strategies for RFD_PEAK_ (CV = 7.52–19.38%), RFD_AVE_ (CV = 9.83–19.42%), and RFD_INDEX_ (CV = 14.78–27.66%), suggesting that RFD performance expression is highly variable across CCT set strategies. All CCT set strategies demonstrated non-significant RMB and 95% LoA values. The SWC values were larger than the SEM values for all CCT set strategies for J_PROP_, MF and t_PROP_. The majority of CCT set strategies showed SWC values lower than SEM values for PF, RFD_PEAK_, RFD_AVE_, and RFD_INDEX_.

### 3.2. Squat Jump

Descriptive statistics for the first and second jump of the BASELINE set of SJs for all CCT set strategies, alongside within- and between-participant PES metrics for each dependent variable, are provided in Table 5. Correlation coefficients indicate that, across all CCT set strategies, between-participant performance expression is stable for J_PROP_, PF, MF, and t_PROP_ (*r* = 0.80–0.99). However, not all CCT set strategies facilitated stable performance expression for RFD_AVE_ (*r* = 0.60–0.97), RFD_50_ (*r* = 0.76–0.93), RFD_100_ (*r* = 0.57–0.91) and RFD_150_ (*r* = 0.55–0.89). All CCT set strategies demonstrated moderate to nearly perfect ICCs across all dependent variables (ICC_3,1_ = 0.55–0.99).

Regarding within-participant PES, all CCT set strategies demonstrated acceptable CV values for J_PROP_, PF, MF, RFD_AVE_ and t_PROP_ (CV = 1.18–9.98%), suggesting acceptable within-participant PES. Unacceptable CV values were observed under nearly all CCT set strategies for RFD_50_ (CV = 12.88–19.56%), RFD_100_ (CV = 9.46–18.53%), and RFD_150_ (CV = 10.04–16.71%). All CCT set strategies demonstrated non-significant RMB and 95% LoA values across all dependent variables. The SWC values were larger than the SEM values for all CCT set strategies for J_PROP_, PF, MF, and t_PROP_. The majority of CCT set strategies showed SWC values lower than SEM values for RFD_AVE_, RFD_50_, RFD_100_, and RFD_150_.

## 4. Discussion

This study aimed to assess the PES of 16 alternative CCT set strategies that vary the TCRP and the organization of rest within CCT prescriptions using RR to enhance within-set vertical jump propulsion. To the authors’ knowledge, this is the first study to assess within- and between-participant PES for this endeavor. The use of PES metrics facilitates a comprehensive evaluation of the consistency of propulsive force–time characteristics across CCT set strategies, both within and between participants. These results can be combined with Houlton et al. [17,18,19] to help practitioners make informed decisions about exercise prescription by balancing the practical effects and PES of different CCT set structures.

For both CMJ and SJ, moderate to nearly perfect ICC_3,1_ values were observed for all CCT set strategies, suggesting that performance expression between participants is stable and systematic bias is acceptable. All set strategies demonstrated acceptable correlations for J_PROP_, PF, MF and t_PROP_. However, RFD variables showed unacceptable correlations (<0.80) with some CCT set strategies, indicating that these strategies do not consistently facilitate stable expression of RFD between participants. Similarly, all CCT set strategies demonstrated acceptable CVs (<10%) and SWC values exceeding the SEM for J_PROP_, PF, MF, and t_PROP_, suggesting that within-participant variance in PES is acceptable and, subsequently, that real changes are detectable in these metrics. As with between-participant PES results, RFD metrics demonstrated unacceptable CVs and SWC values smaller than SEMs, suggesting that high within-participant variance in PES makes it difficult to detect real, meaningful changes in RFD. For all set configurations and metrics, RMBs were non-significant, indicating that CCT set strategies do not produce systematic changes in performance at the group level. However, the varied width of 95% LoAs around RMBs highlight the individualized nature of PAPE-based protocols with some CCT set strategies yielding highly individualized responses (i.e., wide 95% LoA), while others yielded more consistent group-level performance (i.e., narrow 95% LoA). Regardless of the magnitude of variance, non-significant RMBs indicate that at the group level, changes in performance are nuanced, highlighting the need for individualized prescriptions. The results suggest that combining CCT prescriptions with RR facilitates stable performance expression in J_PROP_, PF, MF, and t_PROP_. However, the PES of RFD metrics is less consistent. Thus, practitioners may balance the intervention PES from this study with the effectiveness of CCT set strategies established in our previous studies when prescribing CCT training for moderately strong recreational athletes.

This study introduced a novel approach for assessing the PES of CCT set strategies alongside interpretations of pairwise or means-based analyses. Training methods like CCT often result in non-significant pairwise and means-based effects because of the highly individualized nature of phenomena like PAPE [26,27] and the varied, noisy nature of performance measures like RFD [23,24,25]. Consequently, it becomes difficult to determine whether CCT-based training prescriptions result in true performance improvements at the individual and group levels. We surmised that this secondary assessment of alternative CCT set strategies quantifies within- and between-participant PES, thereby complementing means-based inferential statistics. This combined approach may demonstrate the likelihood of effective acute CCT-based performance enhancement being repeated across multiple exposures, which is characteristic of a typical strength and power training session (i.e., multiple sets of prescribed exercises are completed within one session).

Furthermore, consistently effective performance of CCT prescriptions facilitates accurate monitoring and better determination of augmented performance over time. This may allow practitioners to more accurately predict long-term responses to CCT-based mesocycles. Practitioners can balance pairwise effects with intervention PES to establish the performance potential of CCT set strategies for acutely enhancing vertical jump propulsion. They can do so in the context of the specific characteristics they are targeting (i.e., impulse, maximal force or explosive force application), which may help practitioners individualize training for athletes by accounting for individual sensitivity to PAPE-based protocols. For example, practitioners may prioritize CCT set strategies with high within-participant PES (i.e., low CVs and SEMs smaller than SWC) to individually control dose and response, thereby maximizing the likelihood of observing individual performance enhancement in non-responders to training stimuli. Conversely, for inherent responders, practitioners may consider using CCT set strategies that yield larger effects and demonstrate high between-participant PES to ensure acute performance enhancement at the group level.

Between-participant PES analyses were used in this study to assess the stability with which a variety of TCRPs, combined with RR strategies within CCT prescriptions, facilitate changes in propulsive force expression, as measured by force–time metrics. For both the CMJ and SJ protocols, nearly all CCT set configurations yielded ICCs ranging from large to nearly perfect and strong, significant correlations for J_PROP_, PF, MF, and t_PROP_ (Table 4 and Table 5). High ICC_3,1_ values, observed in both traditional and alternative CCT set strategies, indicate that performance expression was stable and likely to remain stable within groups of athletes outside the sampled groups. This suggests that when aggregated, acute performance expression within CCT set strategies is a stable and repeatable characteristic when targeting these propulsion metrics. However, 95% CIs for these metrics demonstrate that ICCs range from small to nearly perfect between individuals, providing a very wide range of plausible ICC values. Thus, where possible, between-participant PES should be combined with within-participant PES metrics to understand how individual variance affects between-participant indices. At the group level, combining CCT prescriptions with RR does not diminish the consistency of performances, reinforcing their use for performance maintenance or enhancement during combined strength and power training in time-limited team-based training scenarios [17,18,19]. However, individual responses should be considered if training time and density is not a priority.

Between-participant PES of force–time metrics (i.e., RFD variants) was less consistent, as evidenced by a broader range of ICC_3,1_ values, lower correlation coefficients and wider confidence intervals for these metrics than for impulse-, force-, and time-derived metrics. This is consistent with the current understanding of the biological and processing noise associated with RFD [23,24,25]. In the present study, biological noise may be explained by the moderate relative strength levels of the samples used in the cross-sectional studies (Table 1). It has been demonstrated that stronger athletes have higher ratios of type II to type I muscle fibers than weaker athletes [38], limiting weaker athletes’ ability to rapidly and consistently produce propulsive force. In the present study, between-participant PES results demonstrate that PAPE-based RFD expression within CCT prescriptions is highly individualized. The magnitude of CA-induced PAPE is dependent on the net product of individualized neuromuscular excitation and peripheral fatigue profiles [1,2,3]. Thus, the participants recruited for the cross-sectional studies may have been insufficiently strong to consistently observe PAPE responses in RFD metrics, as muscle architecture and individualized excitation and fatigue profiles may have limited the ability of the conditioning activity to enhance synchronous, rapid recruitment of high-threshold motor units [6,67]. In addition to the potential physiological characteristics of the samples, RFD metrics have demonstrated high levels of measurement noise [23,24,25]. Measurement noise is a result of small errors in the force signal and timing of force onset [68]. Signal errors may then be amplified when the force–time curve is differentiated to calculate RFD metrics [69]. This is especially apparent in early-phase RFDs, where movement artefact and signal noise are more prominent. To limit this, the starting threshold used for the propulsive phase for CMJ and SJ was considered to have started when the force signal exceeded 50 N as this threshold has demonstrated acceptable within- and between-participant variability compared to other starting thresholds [25]. This conservative approach aimed to limit the signal noise and movement artefact frequently observed in the early phases of CMJ and SJ propulsion.

While not entirely consistent, CCT set strategies employing longer TCRPs produced higher ICCs and stronger correlations, suggesting that, regardless of RR strategy, longer TCRPs may yield more consistent performance. The increased ICRP between the conditioning activity and explosive activity may contribute to more consistent, rapid application of propulsive force by facilitating better recovery from conditioning activity-induced neuromuscular fatigue [6]. Practitioners can use between-participant PES to prescribe alternative CCT set strategies to consistently maintain or augment the application of vertical jump propulsive force across athletes, while accounting for total training time and training density. However, if RFD is the targeted outcome of a CCT prescription, practitioners may need to prescribe CCT set strategies on an individual basis to enhance their effect.

When considering within-participant PES, the results present a similar picture. All CCT set strategies demonstrated acceptable CVs for J_PROP_, PF, MF and t_PROP_ (Table 4 and Table 5), suggesting that CCT prescriptions consistently facilitate stable individual performance expression of vertical jump propulsive force–time metrics. Furthermore, the evaluation of SWC values relative to corresponding SEM values suggests that meaningful changes in these metrics’ performance are practically detectable [29,30]. However, as with between-participant PES indices, RFD metrics consistently showed higher CVs and larger SEMs than SWC values, suggesting that biological variance and measurement noise may affect PES and limit the detection of meaningful changes. Furthermore, the varied width of the 95% LoA around RMB values demonstrates that some CCT set strategies may yield highly individualized responses (i.e., wide 95% LoA), whereas others yield more consistent group-level performance (i.e., narrower 95% LoA). Thus, despite RMBs suggesting that CCT set strategies do not elicit systematic changes in vertical jump propulsion, they may facilitate nuanced changes in performance at the individual level. However, as *n* < 40, it should also be noted that the sample sizes may limit the stability of 95% LoA values [31]. The combination of high within-participant variability, the highly individualized nature of PAPE, and high measurement noise may compound to limit the stability and detectability of meaningful changes in RFD expression for the majority of CCT set strategies. Therefore, PES between trials may be highly individualized and further justifies experimentation on an individual basis to facilitate understanding of nuanced changes in the magnitude, direction, and variance of the effect.

The results of this study demonstrate that assessing the PES of alternative CCT set strategies may be useful for impulse-, force-, and time-derived metrics in both individual- and team-based training settings. High between-participant PES values indicate that group responses are likely to reflect underlying trends rather than random ordering, given the variance and systematic bias [28,31]. In individual settings, the combined use of within- and between-participant PES may inform individualized programming decisions. When within- and between-participant performance expression is stable, and meaningful changes are detectable, practitioners may confidently prescribe CCT set strategies that elicit true physiological adaptations. This may be particularly valuable when programming training and monitoring training load across strength-focused mesocycles that target J_PROP_, PF, MF, and t_PROP_. However, the sensitivity of this approach depends on the magnitude of SEM and the heterogeneity of the force–time metric. Thus, the large SEMs relative to SWC and the 95% LoA of varying widths observed for RFD variants suggest that using CCT set strategies to target RFD enhancement may be inappropriate given the unpredictably high levels of measurement noise and variability. This may be further pronounced by the sample sizes. As such, practitioners may need to experiment at the individual level to identify the most effective CCT prescriptions to enhance RFD expression during power-based mesocycles.

This secondary analysis was not without limitations. Firstly, the moderately strong, homogeneous samples, while providing high external validity for many male sporting populations, limit the generalizability of the results to other populations, such as females, youth athletes, and elite athletes, who likely possess different potentiation and fatigue profiles [6]. Secondly, the high variability and measurement noise associated with RFD metrics, as evidenced by large CVs and high SEMs, limit the potential of CCT set strategies to elicit PAPE-based performance improvements in these metrics. This may have been further compounded by the sample sizes recruited for the cross-sectional studies (*n* = 14–17). While the sample sizes exceeded statistical power analyses to detect large point estimate ICCs, the relatively smaller sample sizes may have contributed to the wide CIs observed around stability metrics [70], thus reducing the precision estimation [71]. Finally, secondary analyses of the CCT set strategies used in cross-sectional studies provide detailed insight into within-session PES of individual and group athletes. However, between-session PES has not been considered, which would benefit practitioners in predicting outcomes of strength- and power-focused mesocycles employing CCT methods. Future research should consider PES across multiple sessions using larger samples. Furthermore, a broader range of athlete groups with greater relative strength levels should be considered alongside pairwise effects in these populations to enhance the generalizability and practical application of PES analyses.

## 5. Conclusions

Our study used a novel secondary analysis to assess the PES of 16 alternative CCT set strategies for enhancing vertical jump propulsion. The results demonstrated that all 16 set configurations achieved high within-session PES for key propulsion metrics, including J_PROP_, PF, MF, and t_PROP_. However, the highly individualized and noisy nature of force–time-derived metrics, such as RFD, led to less consistent within-session PES. This is highlighted by an analysis of within-participant stability metrics, in which acceptable CVs and large SWCs relative to SEMs indicate that practically meaningful changes in J_PROP_, PF, MF, and t_PROP_ expression are stable and detectable, whereas the opposite holds for RFD metrics. Practitioners may balance the PES established in the present study with the pairwise and pooled effects established in our recent cross-sectional studies [17,18,19] to establish the performance potential of the alternative CCT set strategies assessed across this series of studies. Practitioners can use these strategies to facilitate stable expression of key metrics such as J_PROP_, PF, MF, and t_PROP_ in group-based training environments with time constraints. For power-focused mesocycles targeting RFD, individualized programming may be necessary to account for within-participant variability and noise.

## Figures and Tables

**Table 1:** Sample demographic information for all three analyzed studies. All data are presented as mean ± SD.

	Houlton et al. [17] (*n* = 14)	Houlton et al. [18] (*n* = 17)	Houlton et al. [19] (*n* = 15)
Age (years)	24.90 ± 4.50	25.50 ± 3.00	26.00 ± 2.60
Stature (m)	1.76 ± 0.06	1.76 ± 0.07	1.75 ± 0.08
Body mass (kg)	81.84 ± 10.25	84.25 ± 5.51	82.49 ± 5.32
Back squat 3RM (kg)	131.71 ± 16.32	139.88 ± 12.87	141.33 ± 13.64
Relative strength	1.63 ± 0.14	1.66 ± 0.13	1.72 ± 0.15

3RM, three-repetition maximum. Relative strength is calculated as back squat 3RM divided by body mass.

**Table 2:** The independent variables of the three cross-sectional studies.

	IV1			IV2
Study	TCRP * (s)	ICRP (s)	RR ^†^ (s)	Vertical Jumps
Houlton et al. [17]	0	0	0	CMJ SJ
	60	60	0	
	120	120	0	
	180	180	0	
	240	240	0	
	300	300	0	
Houlton et al. [18]	60	30	30	CMJ SJ
	120	90	30	
	180	150	30	
	240	210	30	
	300	270	30	
Houlton et al. [19]	60	0	60	CMJ SJ
	120	60	60	
	180	120	60	
	240	180	60	
	300	240	60	

CMJ, countermovement jump; ICRP, intra-contrast rest period; IV, independent variable; RR, rest redistribution; SJ, squat jump; TCRP, total contrast rest period. * TCRP = ICRP + RR. ^†^ RR split equally between conditioning activity repetitions.

**Table 3:** The dependent variables of the three cross-sectional studies.

Countermovement Jump	Squat Jump
Propulsive Impulse (N∙s)	Propulsive Impulse (N∙s)
Peak Force (N)	Peak Force (N)
Mean Force (N)	Mean Force (N)
Peak RFD (N∙s^−1^)	Average RFD (N∙s^−1^)
Average RFD (N∙s^−1^)	RFD 50 ms (N∙s^−1^)
RFD Index (N∙s^−2^)	RFD 100 ms (N∙s^−1^)
Propulsion time (s)	RFD 150 ms (N∙s^−1^)
	Propulsion time (s)

RFD, rate of force development.

**Table 4:** Performance expression stability (PES) statistics for total complex rest period (TCRP) and countermovement jump protocols. Between-participant PES is estimated using Pearson’s correlation coefficient (*r*) with 95% confidence interval (95% CI) and intra-class correlation coefficient (ICC_3,1_) with 95% CI. Within-participant PES is estimated using relative mean bias (RMB) with 95% limit of agreement (95% LoA), coefficient of variation (CV) with 95% CI, standard error of measure (SEM) with 95% CI and smallest worthwhile change (SWC) with 95% CI.

Variable	Study	TCRP (s)	R1	R2	Between Participant PES		Within Participant PES			
					*r* (95% CI)	ICC_3,1_ (95% CI)	RMB (95% LoA)	CV (95% CI)	SEM (95% CI)	SWC (95% CI)
Propulsive Impulse (N∙s)	1	0	219.39 ± 39.12	214.00 ± 42.99	0.95 (0.85, 0.99)	0.95 (0.85, 0.98)	5.39 (−20.59, 31.37)	3.83 (1.80, 5.86)	9.37 (6.80, 5.10)	14.15 (−7.03, 35.32)
		60	218.42 ± 44.71	216.80 ± 47.25	0.95 (0.85, 0.99)	0.95 (0.86, 0.99)	1.62 (−26.59, 29.84)	3.43 (2.24, 4.62)	10.18 (7.38, 16.40)	15.80 (−7.84, 39.45)
		120	219.11 ± 37.20	219.63 ± 31.86	0.95 (0.85, 0.99)	0.95 (0.84, 0.98)	−0.52 (−23.85, 22.82)	2.92 (1.70, 4.14)	8.42 (6.10, 13.56)	11.90 (−5.91, 29.70)
		180	218.98 ± 38.62	220.58 ± 34.11	0.95 (0.85, 0.99)	0.95 (0.85, 0.98)	−1.59 (−25.32, 22.13)	3.07 (1.65, 4.49)	8.56 (6.20, 13.79)	12.52 (−6.22, 31.25)
		240	226.63 ± 49.14	231.39 ± 43.69	0.98 (0.93, 0.99)	0.97 (0.92, 0.99)	−4.76 (−26.33, 16.81)	2.70 (1.18, 4.21)	7.78 (5.64, 12.54)	15.99 (−7.94, 39.92)
		300	228.65 ± 36.29	231.00 ± 37.49	0.95 (0.84, 0.98)	0.95 (0.85, 0.98)	−2.35 (−25.61, 20.91)	2.60 (1.07, 4.13)	8.39 (6.08, 13.52)	12.68 (−6.30, 31.65)
	2	60	219.49 ± 31.77	221.60 ± 27.63	0.93 (0.81, 0.98)	0.93 (0.81, 0.97)	−4.02 (−25.87, 17.84)	2.28 (0.50, 4.05)	7.88 (5.87, 12.00)	10.17 (−5.05, 25.38)
		120	225.46 ± 21.83	223.62 ± 22.30	0.97 (0.91, 0.99)	0.97 (0.91, 0.99)	0.85 (−11.91, 13.61)	1.56 (0.93, 2.19)	4.60 (3.43, 7.00)	8.69 (−4.31, 21.69)
		180	223.98 ± 21.50	221.12 ± 23.67	0.94 (0.83, 0.98)	0.93 (0.82, 0.98)	−0.06 (−17.59, 17.46)	1.84 (1.02, 2.66)	6.32 (4.71, 9.62)	8.13 (−4.04, 20.29)
		240	223.75 ± 23.41	225.74 ± 23.91	0.98 (0.94, 0.99)	0.98 (0.94, 0.99)	−2.27 (−12.09, 7.55)	1.12 (0.77, 1.46)	3.54 (2.64, 5.39)	7.94 (−3.94, 19.82)
		300	226.46 ± 25.44	222.57 ± 25.14	0.94 (0.84, 0.98)	0.94 (0.85, 0.98)	3.06 (−14.04, 20.16)	1.81 (0.64, 2.98)	6.17 (4.60, 9.39)	8.81 (−4.37, 21.99)
	3	60	220.51 ± 21.10	221.48 ± 23.39	0.97 (0.91, 0.99)	0.97 (0.90, 0.99)	−1.43 (−12.40, 9.55)	1.52 (0.98, 2.06)	3.96 (2.90, 6.25)	7.40 (−3.67, 18.46)
		120	222.40 ± 16.25	223.01 ± 17.58	0.95 (0.85, 0.98)	0.95 (0.85, 0.98)	−1.31 (−11.88, 9.27)	1.42 (0.99, 1.84)	3.82 (2.79, 6.02)	5.64 (−2.80, 14.08)
		180	222.97 ± 21.21	222.18 ± 21.77	0.97 (0.90, 0.99)	0.97 (0.90, 0.99)	−0.14 (−11.50, 11.23)	1.36 (0.82, 1.90)	4.10 (3.00, 6.47)	7.39 (−3.64, 18.43)
		240	223.48 ± 22.34	222.78 ± 20.74	0.94 (0.83, 0.98)	0.95 (0.85, 0.98)	0.14 (−14.10, 14.38)	1.85 (1.15, 2.55)	5.14 (3.76, 8.10)	7.30 (−3.64, 18.22)
		300	223.82 ± 17.50	223.04 ± 19.33	0.97 (0.92, 0.99)	0.97 (0.91, 0.99)	0.80 (−8.13, 9.74)	1.22 (0.83, 1.62)	3.22 (2.36, 5.08)	6.11 (−3.03, 15.26)
Peak Force (N)	1	0	2037.48 ± 339.58	2066.64 ± 349.30	0.93 (0.78, 0.98)	0.93 (0.80, 0.98)	−29.16 (−285.14, 226.81)	3.34 (2.03, 4.65)	92.35 (66.95, 148.78)	118.42 (−58.81, 295.66)
		60	1977.35 ± 297.83	2057.81 ± 345.11	0.88 (0.65, 0.96)	0.87 (0.64, 0.96)	−80.46 (−405.60, 244.68)	3.30 (0.61, 5.99)	117.30 (85.04, 188.98)	111.63 (−55.44, 278.71)
		120	1967.95 ± 287.03	1985.82 ± 236.02	0.91 (0.75, 0.97)	0.90 (0.72, 0.97)	−17.88 (−251.33, 215.58)	2.97 (1.49, 4.44)	84.22 (61.06, 135.69)	90.31 (−44.85, 225.46)
		180	1954.97 ± 293.64	2022.75 ± 301.33	0.86 (0.61, 0.96)	0.86 (0.62, 0.95)	−67.79 (−374.36, 238.79)	4.22 (2.28, 6.16)	110.60 (80.18, 178.19)	102.89 (−51.10, 256.86)
		240	2025.78 ± 35.43	2085.72 ± 329.08	0.88 (0.66, 0.96)	0.88 (0.67, 0.96)	−59.94 (−371.79, 241.90)	4.21 (2.42, 6.00)	112.50 (81.56, 181.25)	112.91 (−56.07, 281.89)
		300	2021.57 ± 350.45	2026.16 ± 300.42	0.86 (0.61, 0.96)	0.86 (0.62, 0.95)	−4.59 (−354.57, 345.39)	4.07 (2.31, 5.83)	126.26 (91.53, 203.41)	112.11 (−55.68, 279.89)
	2	60	1926.46 ± 177.06	1996.81 ± 109.69	0.92 (0.80, 0.97)	0.82 (0.58, 0.93)	−47.60 (−245.25, 150.05)	2.83 (1.17, 4.49)	71.31 (53.11, 108.52)	59.09 (−29.34, 147.52)
		120	2016.16 ± 125.65	1998.47 ± 157.40	0.89 (0.59, 0.92)	0.88 (0.59, 0.91)	16.48 (−212.68, 245.64)	3.01 (1.77, 4.26)	82.67 (61.57, 125.82)	59.53 (−29.66, 148.63)
		180	1988.99 ± 183.53	1976.60 ± 131.93	0.81 (0.45, 0.99)	0.81 (0.46, 0.99)	2.35 (−233.93, 238.63)	2.88 (1.53, 4.24)	85.24 (63.49, 129.73)	53.24 (−26.44, 132.92)
		240	2027.15 ± 182.79	2002.85 ± 168.65	0.80 (0.52, 0.92)	0.80 (0.52, 0.92)	41.78 (−204.43, 287.99)	3.21 (1.75, 4.66)	88.83 (66.15, 135.19)	68.28 (−33.91, 170.47)
		300	2015.32 ± 141.57	1951.20 ± 193.35	0.84 (0.61, 0.94)	0.83 (0.58, 0.93)	56.89 (−190.71, 304.49)	3.43 (1.47, 5.39)	89.33 (66.53, 135.95)	74.31 (−36.90, 185.51)
	3	60	1939.61 ± 194.19	1927.23 ± 190.10	0.87 (0.65, 0.96)	0.87 (0.66, 0.96)	22.87 (−162.14, 207.87)	2.77 (1.87, 3.67)	66.74 (48.87, 105.26)	64.30 (−31.93, 160.52)
		120	1935.73 ± 168.29	1984.27 ± 140.64	0.84 (0.58, 0.95)	0.83 (0.56, 0.94)	−42.99 (−216.07, 130.08)	2.98 (2.02, 3.95)	62.44 (45.71, 98.47)	52.34 (−26.00, 130.68)
		180	1970.66 ± 125.66	2011.38 ± 135.41	0.64 (0.19, 0.87)	0.64 (0.21, 0.86)	−35.37 (−274.21, 176.46)	2.76 (1.24, 4.28)	76.42 (55.95, 120.53)	44.18 (−21.94, 110.30)
		240	1947.96 ± 191.15	1944.11 ± 141.17	0.69 (0.27, 0.89)	0.68 (0.27, 0.88)	1.76 (−260.56, 264.07)	3.48 (1.96, 5.00)	94.64 (69.29, 149.25)	55.72 (−27.67, 139.11)
		300	1948.48 ± 141.30	1982.15 ± 138.17	0.70 (0.30, 0.89)	0.70 (0.32, 0.89)	−37.30 (−241.05, 166.44)	3.35 (2.23, 4.46)	73.51 (53.82, 115.93)	46.87 (−23.28, 117.02)
Mean Force (N)	1	0	1590.80 ± 269.24	1565.55 ± 246.06	0.98 (0.95, 0.99)	0.98 (0.94, 0.99)	25.25 (−79.10, 129.60)	2.00 (1.30, 2.69)	37.65 (27.29, 60.65)	88.70 (−44.05, 221.44)
		60	1568.26 ± 228.00	1555.80 ± 235.53	0.98 (0.94, 0.99)	0.98 (0.94, 0.99)	12.47 (−78.36, 103.29)	1.64 (0.90, 2.38)	32.77 (23.75, 52.79)	79.64 (−39.55, 198.84)
		120	1584.12 ± 222.75	1585.81 ± 210.42	0.97 (0.91, 0.99)	0.97 (0.91, 0.99)	−1.69 (−108.02, 104.64)	1.72 (1.03, 2.41)	38.36 (27.81, 61.80)	74.42 (−36.96, 185.79)
		180	1609.61 ± 232.69	1589.44 ± 218.03	0.98 (0.94, 0.99)	0.98 (0.94, 0.99)	20.17 (−69.82, 110.17)	1.42 (0.64, 2.20)	32.47 (23.54, 52.30)	77.53 (−38.50, 193.55)
		240	1612.99 ± 244.01	1600.61 ± 227.64	0.98 (0.95, 0.99)	0.98 (0.94, 0.99)	12.38 (−76.28, 101.03)	1.66 (1.09, 2.22)	31.98 (23.19, 51.53)	81.08 (−40.26, 202.42)
		300	1642.72 ± 286.11	1597.36 ± 233.22	0.97 (0.91, 0.99)	0.95 (0.86, 0.98)	45.36 (−112.61, 203.34)	2.12 (0.86, 3.38)	56.99 (41.32, 91.82)	90.01 (−44.70, 224.72)
	2	60	1585.94 ± 151.74	1585.91 ± 130.95	0.97 (0.90, 0.99)	0.95 (0.87, 0.98)	10.00 (−90.29, 110.29)	1.87 (1.09, 2.65)	36.18 (26.95, 55.07)	56.15 (−27.89, 140.18)
		120	1633.02 ± 144.26	1615.30 ± 152.02	0.96 (0.89, 0.99)	0.96 (0.89, 0.99)	19.41 (−80.63, 119.44)	1.68 (0.90, 2.45)	36.09 (26.88, 54.93)	60.92 (−30.25, 152.09)
		180	1626.46 ± 131.84	1605.42 ± 137.99	0.93 (0.82, 0.98)	0.93 (0.82, 0.97)	16.83 (−82.01, 115.67)	1.69 (0.90, 2.47)	35.66 (26.56, 54.27)	46.79 (−23.24, 116.81)
		240	1620.80 ± 147.42	1625.53 ± 141.16	0.95 (0.85, 0.98)	0.95 (0.86, 0.98)	−1.87 (−104.93, 101.20)	1.81 (1.29, 2.34)	37.18 (27.69, 56.59)	54.89 (−27.26, 137.04)
		300	1621.29 ± 139.92	1596.25 ± 170.51	0.94 (0.85, 0.98)	0.93 (0.81, 0.97)	17.44 (−117.96, 152.84)	2.18 (0.99, 3.37)	48.85 (36.38, 74.34)	61.35 (−30.47, 153.18)
	3	60	1562.77 ± 119.73	1556.77 ± 101.04	0.84 (0.58, 0.95)	0.84 (0.59, 0.94)	8.03 (−113.89, 129.96)	2.16 (1.11, 3.21)	43.99 (32.20, 69.37)	37.01 (−18.38, 92.40)
		120	1571.05 ± 122.29	1581.77 ± 119.74	0.89 (0.70, 0.97)	0.90 (0.72, 0.97)	−8.26 (113.80, 97.29)	2.02 (1.27, 2.78)	38.08 (27.88, 60.05)	40.19 (−19.96, 100.33)
		180	1604.42 ± 127.34	1614.66 ± 111.75	0.89 (0.69, 0.96)	0.89 (0.69, 0.96)	−3.85 (−117.37, 109.67)	1.90 (1.11, 2.70)	40.95 (29.98, 64.59)	40.24 (−19.98, 100.46)
		240	1556.96 ± 131.07	1569.85 ± 116.22	0.95 (0.84, 0.98)	0.94 (0.83, 0.98)	−7.27 (−92.02, 77.49)	1.60 (0.97, 2.22)	30.58 (22.39, 48.22)	41.52 (−20.62, 103.65)
		300	1576.28 ± 131.79	1566.03 ± 158.21	0.94 (0.83, 0.98)	0.93 (0.81, 0.98)	9.74 (−94.91, 114.39)	1.70 (0.85, 2.56)	37.75 (27.64, 59.54)	48.29 (−23.98, 120.55)
Peak RFD (N∙s^−1^)	1	0	6105.00 ± 3287.97	5137.86 ± 2789.76	0.88 (0.64, 0.96)	0.86 (0.63, 0.95)	967.14 (−2161.52, 4095.81)	17.73 (7.08, 28.38)	1128.72 (818.27, 1818.41)	1061.31 (−527.08, 2649.70)
		60	5876.43 ± 2125.98	5652.14 ± 3041.70	0.80 (0.46, 0.93)	0.76 (0.41, 0.92)	224.29 (−3419.23, 3867.80)	18.95 (13.21, 24.70)	1314.53 (952.98, 2117.77)	902.15 (−448.04, 2252.34)
		120	5873.57 ± 1895.18	5174.29 ± 2167.75	0.70 (0.28, 0.90)	0.70 (0.29, 0.89)	699.29 (−2402.49, 3801.06)	17.28 (12.47, 22.09)	1118.93 (811.17, 1802.64)	710.31 (−352.76, 1773.38)
		180	4761.43 ± 2011.92	4740.00 ± 1844.82	0.56 (0.22, 0.80)	0.59 (0.21, 0.80)	21.43 (−3912.38, 3955.21)	17.92 (10.60, 25.24)	1419.15 (1028.82, 2286.32)	662.95 (−329.24, 1655.13)
		240	5610.71 ± 3077.62	6024.29 ± 3449.38	0.84 (0.55, 0.95)	0.84 (0.56, 0.94)	−413.57 (−4137.71, 3310.57)	17.50 (10.20, 24.81)	1343.50 (973.98, 2164.44)	1125.11 (−558.77, 2808.98)
		300	5645.71 ± 3000.88	5942.86 ± 3708.25	0.84 (0.55, 0.95)	0.83 (0.54, 0.94)	−297.14 (−4296.59, 3702.30)	18.09 (12.66, 23.52)	1442.91 (1046.05, 2324.60)	1159.75 (−575.97, 2895.46)
	2	60	5144.29 ± 1658.21	5841.43 ± 1812.47	0.60 (0.16, 0.84)	0.59 (0.18, 0.83)	−542.94 (−3906.99, 2821.11)	17.50 (10.76, 24.24)	1213.67 (903.91, 1847.12)	663.80 (−329.67, 1657.26)
		120	5425.00 ± 2345.16	5372.86 ± 2297.63	0.71 (0.35, 0.89)	0.72 (0.38, 0.89)	104.12 (−3221.19, 3429.42)	19.38 (15.23, 23.52)	1199.58 (893.41, 1825.68)	765.06 (−379.96, 1910.08)
		180	5570.00 ± 1420.15	5657.14 ± 2085.37	0.55 (0.09–0.81)	0.55 (0.11, 0.81)	−207.06 (−3770.63, 3356.52)	18.76 (13.61, 23.90)	1285.69 (957.54, 1956.73)	649.36 (−322.50, 1621.22)
		240	5535.00 ± 2396.80	4579.29 ± 1509.43	0.74 (0.40, 0.90)	0.72 (0.39, 0.89)	659.41 (2526.12, 3844.94)	15.99 (10.56, 21.42)	1149.35 (856.00, 1749.23)	761.32 (−378.10, 1900.75)
		300	5159.29 ± 2608.75	5034.29 ± 2034.42	0.66 (0.27, 0.87)	0.65 (0.27, 0.86)	223.53 (−3307.54, 3754.60)	18.01 (12.04, 23.98)	1273.97 (948.81, 1938.89)	734.47 (−364.77, 1833.71)
	3	60	5513.57 ± 2016.18	5126.43 ± 2282.01	0.94 (0.81, 0.98)	0.92 (0.80, 0.98)	374.00 (−1184.18, 1932.18)	10.72 (6.65, 14.80)	562.14 (411.56, 886.56)	720.46 (−357.81, 1798.73)
		120	4913.57 ± 2256.19	4735.71 ± 1867.18	0.94 (0.82, 0.98)	0.92 (0.78, 0.97)	221.33 (−1431.42, 1874.09)	8.80 (5.77, 11.84)	596.26 (436.54, 940.36)	732.49 (−363.78, 1828.75)
		180	4310.71 ± 1654.50	4386.43 ± 1554.46	0.92 (0.77, 0.97)	0.92 (0.77, 0.97)	−155.33 (−1420.06, 1109.39)	7.52 (4.75, 10.30)	456.27 (334.05, 719.59)	546.48 (−271.40, 1364.36)
		240	4665.00 ± 1667.78	4140.57 ± 1968.52	0.85 (0.60, 0.95)	0.84 (90.58, 0.94)	508.80 (−1478.11, 2495.71)	15.85 (6.60, 25.11)	716.82 (524.80, 1130.49)	618.53 (−307.18, 1544.24)
		300	4385.00 ± 1649.30	4107.86 ± 1537.35	0.90 (0.72, 0.97)	0.90 (0.73, 0.97)	256.00 (−1168.84, 1680.84)	12.04 (6.12, 17.97)	514.04 (376.34, 810.69)	556.51 (−276.38, 1389.41)
Average RFD (N∙s^−1^)	1	0	2809.72 ± 1481.53	2852.79 ± 1631.79	0.87 (0.63, 0.96)	0.88 (0.66, 0.96)	−43.07 (−1621.86, 1535.71)	19.40 (5.19, 33.62)	569.58 (412.92, 917.61)	535.32 (−265.86, 1336.50)
		60	2458.74 ± 1139.34	2749.16 ± 1169.39	0.70 (0.27, 0.90)	0.70 (0.29, 0.89)	−290.42 (−2040.64, 1459.80)	19.42 (9.71, 29.13)	631.42 (457.75, 1017.25)	399.87 (−198.59, 998.33)
		120	2352.39 ± 954.91	2063.84 ± 1163.51	0.73 (0.32, 0.91)	0.71 (0.32, 0.90)	288.55 (−1290.14, 1867.24)	17.76 (8.86, 26.66)	569.54 (412.89, 917.56)	369.15 (−183.33, 921.64)
		180	1898.97 ± 817.70	2167.69 ± 1173.91	0.67 (0.21, 0.88)	0.62 (0.16, 0.86)	−268.72 (−1987.27, 1449.83)	19.37 (11.76, 26.99)	620.00 (449.47, 998.84)	350.73 (−174.19, 875.65)
		240	2971.48 ± 1784.49	2881.27 ± 1685.56	0.87 (0.63, 0.96)	0.88 (0.66, 0.96)	90.20 (−1648.35, 1828.76)	15.24 (7.41, 23.07)	627.22 (454.70, 1010.47)	596.37 (−296.18, 1488.91)
		300	2352.24 ± 1454.23	2733.62 ± 1898.95	0.96 (0.87, 0.99)	0.92 (0.78, 0.98)	−381.38 (−1678.81, 916.05)	16.51 (12.15, 20.87)	468.07 (339.33, 754.09)	584.84 (−290.45, 1460.14)
	2	60	2091.33 ± 676.18	2076.14 ± 754.08	0.66 (0.25, 0.86)	0.67 (0.29, 0.86)	−21.91 (−1159.84, 1116.02)	15.38 (11.38, 19.37)	410.53 (305.75, 624.80)	238.54 (−118.47, 595.55)
		120	2599.52 ± 1211.61	2670.18 ± 1264.41	0.83 (0.58, 0.94)	0.84 (0.61, 0.94)	59.12 (−1283.91, 1402.15)	13.60 (10.60, 16.59)	484.52 (360.86, 737.41)	405.73 (−201.50, 1012.96)
		180	2269.15 ± 843.90	2345.21 ± 955.18	0.72 (0.36, 0.89)	0.72 (0.37, 0.89)	−126.68 (−1352.52, 1099.17)	16.44 (9.76, 23.11)	442.25 (329.37, 673.07)	285.08 (−141.58, 711.74)
		240	2340.72 ± 824.07	2264.52 ± 816.07	0.61 (0.18, 0.84)	0.62 (0.22, 0.85)	95.88 (−1214.66, 1406.42)	17.32 (11.98, 22.66)	472.80 (352.13, 719.57)	261.59 (−129.92, 653.10)
		300	2416.82 ± 1122.13	2117.13 ± 724.67	0.73 (0.38, 0.90)	0.68 (0.31, 0.87)	352.72 (−1077.59, 1783.03)	16.77 (10.51, 23.03)	516.01 (384.31, 785.33)	320.05 (−158.95, 799.04)
	3	60	2038.97 ± 737.41	2075.42 ± 748.55	0.77 (0.42, 0.92)	0.78 (0.46, 0.92)	−17.62 (−1065.61, 1030.37)	14.97 (9.74, 20.20)	378.08 (276.80, 596.27)	268.50 (−133.35, 670.36)
		120	2096.20 ± 770.69	2197.33 ± 676.98	0.79 (0.47, 0.93)	0.76 (0.43, 0.91)	6.39 (−1145.33, 1158.10)	14.08 (9.44, 18.72)	415.50 (304.20, 655.29)	284.31 (−141.20, 709.82)
		180	2197.50 ± 830.07	2164.13 ± 887.20	0.72 (0.32, 0.90)	0.72 (0.36, 0.90)	−27.29 (−1301.39, 1246.81)	18.18 (9.59, 26.77)	459.66 (336.53, 724.92)	294.14 (−146.08, 734.36)
		240	2117.77 ± 976.00	2087.52 ± 814.36	0.85 (0.59, 095)	0.85 (0.62, 0.95)	−44.75 (−1041.03, 951.51)	14.00 (8.24, 19.76)	359.42 (263.14, 566.85)	313.54 (−155.71, 782.79)
		300	2152.71 ± 590.16	2043.37 ± 682.18	0.88 (0.67, 0.96)	0.86 (0.63, 0.95)	51.67 (−710.40, 813.74)	9.83 (5.72, 13.94)	274.93 (201.28, 433.59)	244.18 (−121.27, 609.64)
RFD index (N∙s^−2^)	1	0	47,171.02 ± 34,195.80	34,868.79 ± 19,136.17	0.90 (0.70, 0.97)	0.76 (0.42, 0.92)	12,302.22 (−24,878.87, 49,348.32)	20.17 (11.14, 29.20)	13,412.68 (9723.58, 21,608.40)	9766.03 (−4850.16, 24,382.22)
		60	34,849.43 ± 12,792.04	34,410.04 ± 19,274.14	0.76 (0.38, 0.92)	0.71 (0.32, 0.90)	439.40 (−24,557.72, 25,439.51)	20.48 (12.03, 28.93)	9018.31 (6537.86, 14,528.89)	5618.61 (−2790.40, 14,027.63)
		120	40,344.08 ± 27,558.07	32,494.69 ± 18,797.18	0.75 (0.36, 0.92)	0.70 (0.29, 0.89)	7849.39 (−28,129.75, 43,828.52)	26.61 (17.12, 36.09)	12,980.75 (9410.45, 20,912.55)	8221.33 (−4083.00, 20,525.66)
		180	44,767.97 ± 30,494.19	34,832.89 ± 19,475.00	0.90 (0.71, 0.97)	0.82 (0.52, 0.94)	9935.08 (−20,389.04, 40,259.20)	19.33 (11.38, 27.28)	10,940.75 (7931.54, 17,626.01)	8963.91 (−4451.80, 22,379.62)
		240	31,508.50 ± 11,138.66	28,579.97 ± 12,974.23	0.60 (0.11, 0.86)	0.60 (0.12, 0.85)	2928.54 (−18,369.445, 24,226.52)	21.81 (15.45, 28.17)	7683.75 (5570.36, 12,378.85)	4185.52 (−2078.68, 10,449.72)
		300	61,499.61 ± 98,186.86	42,524.38 ± 36,723.43	0.93 (0.79, 0.98)	0.61 (0.14, 0.86)	18,975.24 (−10,9381.22, 14,731.70)	20.30 (11.23, 29.37)	46,303.35 (33,567.79, 74,596.66)	25,682.65 (−12,754.92, 64,120.22)
	2	60	34,731.59 ± 12,263.08	34,059.84 ± 15,122.50	0.72 (0.37, 0.89)	0.73 (0.39, 0.89)	4757.75 (−61,909.90, 71,425.39)	21.64 (15.99, 27.29)	24,052.03 (17,913.22, 36,605.48)	17,390.99 (−8636.98, 43,418.97)
		120	39,624.95 ± 14,812.40	35,613.82 ± 17,944.64	0.83 (0.58, 0.94)	0.82 (0.56, 0.93)	4404.27 (−17,075.72, 25,884.25)	21.65 (14.91, 28.38)	7749.19 (5771.37, 11,793.72)	6254.08 (−3106.00, 15,614.16)
		180	37,985.17 ± 13,440.82	34,405.85 ± 15,487.98	0.74 (0.40, 0.90)	0.73 (0.41, 0.90)	2817.71 (−16,832.87, 22,468.30)	16.89 (9.97, 23.81)	7089.43 (5279.99, 10,789.61)	4767.58 (−2367.75, 11,902.92)
		240	40,969.06 ± 27,574.50	34,291.83 ± 24,829.77	0.76 (0.43, 0.91)	0.75 (0.44, 0.90)	5781.85 (−27,302.66, 38,866.36)	18.43 (10.46, 26.40)	11,937.34 (8890.57, 18,167.78)	8312.79 (−4128.43, 20,754.01)
		300	51,539.24 ± 66,327.84	48,531.08 ± 55,868.22	0.91 (0.76, 0.97)	0.97 (0.91, 0.99)	8953.47 (−40,518.34, 58,425.30)	18.68 (11.82, 25.54)	10,634.85 (7920.51, 16,185.48)	19,482.76 (−9675.83, 48,641.35)
	3	60	47,626.43 ± 47,976.20	37,854.67 ± 25,761.64	0.91 (0.76, 0.97)	0.77 (0.45, 0.92)	7692.06 (−39,302.50, 54,686.62)	18.78 (10.63, 26.93)	17,846.57 (13,065.95, 28,145.81)	12,945.38 (−6429.14, 32,319.90)
		120	45,439.15 ± 31,311.45	46,025.60 ± 27,506.39	0.83 (0.56, 0.94)	0.84 (0.58, 0.94)	−405.51 (−33,628.15, 32,817.12)	16.40 (9.24, 23.56)	11,987.49 (8776.36, 18,905.46)	9894.12 (−4913.77, 24,702.01)
		180	35,303.79 ± 13,759.79	36,812.39 ± 11,697.23	0.73 (0.34, 0.90)	0.73 (0.36, 0.90)	−1641.24 (−19,927.32, 16,644.84)	16.93 (12.50, 21.35)	6596.97 (4829.82, 10,404.07)	4287.60 (−2129.38, 10,704.58)
		240	31,386.41 ± 10,362.96	26,415.82 ± 9716.29	0.74 (0.36, 0.91)	0.73 (0.37, 0.90)	3848.58 (−11,671.47, 19,368.62)	14.78 (8.50, 21.06)	5615.16 (4111.01, 8855.66)	3774.49 (−1874.55, 9423.52)
		300	30,833.98 ± 9625.70	29,164.69 ± 11,193.62	0.75 (0.38, 0.91)	0.72 (0.35, 0.90	262.99 (−19,310.36, 19,836.35)	17.19 (12.50, 21.88)	7061.16 (5169.66, 11,136.15)	4459.42 (−2214.71, 11,133.55)
Propulsion Time (s)	1	0	0.29 ± 0.05	0.29 ± 0.04	0.93 (0.78, 0.98)	0.91 (0.74, 0.97)	0.00 (−0.04, 0.04)	3.60 (2.28, 4.92)	0.01 (0.01, 0.02)	0.02 (−0.01, 0.04)
		60	0.29 ± 0.04	0.29 ± 0.04	0.96 (0.88, 0.99)	0.96 (0.88, 0.99)	0.00 (−0.03, 0.02)	2.42 (1.57, 3.26)	0.01 (0.01, 0.01)	0.01 (−0.01, 0.03)
		120	0.29 ± 0.04	0.29 ± 0.04	0.94 (0.82, 0.98)	0.94 (0.83, 0.98)	0.00 (−0.03, 0.03)	2.77 (1.80, 3.74)	0.01 (0.01, 0.02)	0.01 (−0.01, 0.04)
		180	0.28 ± 0.05	0.29 ± 0.05	0.99 (0.95, 1.00)	0.99 (0.95, 1.00)	−0.01 (−0.02, 0.01)	2.26 (1.31, 3.21)	0.01 (0.00, 0.01)	0.02 (−0.01, 0.04)
		240	0.29 ± 0.04	0.30 ± 0.04	0.86 (0.60, 0.95)	0.86 (0.62, 0.95)	−0.01 (−0.05, 0.03)	3.48 (1.22, 5.74)	0.02 (0.01, 0.03)	0.01 (−0.01, 0.04)
		300	0.28 ± 0.04	0.30 ± 0.04	0.80 (0.46, 0.93)	0.80 (0.48, 0.93)	−0.02 (−0.07, 0.03)	5.03 (1.80, 8.27)	0.02 (0.01, 0.03)	0.01 (−0.01, 0.04)
	2	60	0.28 ± 0.03	0.29 ± 0.03	0.91 (0.76, 0.97)	0.91 (0.77, 0.97)	−0.01 (−0.04, 0.03)	3.05 (1.95, 4.14)	0.01 (0.01, 0.02)	0.01 (−0.01, 0.03)
		120	0.28 ± 0.03	0.28 ± 0.04	0.83 (0.57, 0.94)	0.83 (0.59, 0.93)	0.00 (−0.04, 0.04)	3.49 (1.74, 5.24)	0.01 (0.01, 0.02)	0.01 (−0.01, 0.03)
		180	0.28 ± 0.04	0.28 ± 0.04	0.94 (0.84, 0.98)	0.94 (0.84, 0.98)	0.00 (−0.03, 0.02)	2.59 (1.40, 3.78)	0.01 (0.01, 0.02)	0.01 (−0.01, 0.04)
		240	0.28 ± 0.04	0.28 ± 0.03	0.87 (0.67, 0.95)	0.88 (0.70, 0.95)	0.00 (−0.04, 0.04)	3.84 (2.84, 4.84)	0.01 (0.01, 0.02)	0.01 (−0.01, 0.03)
		300	0.28 ± 0.04	0.29 ± 0.04	0.91 (0.77, 0.97)	0.90 (0.75, 0.96)	0.00 (−0.04, 0.03)	3.28 (1.94, 4.62)	0.01 (0.01, 0.02)	0.01 (−0.01, 0.03)
	3	60	0.29 ± 0.03	0.30 ± 0.04	0.90 (0.73, 0.97)	0.90 (0.74, 0.97)	−0.01 (−0.04, 0.02)	3.76 (2.39, 5.12)	0.01 (0.01, 0.02)	0.01 (−0.01, 0.03)
		120	0.28 ± 0.03	0.29 ± 0.04	0.91 (0.75, 0.97)	0.91 (0.76, 0.97)	−0.01 (−0.04, 0.02)	4.24 (3.19, 5.30)	0.01 (0.01, 0.02)	0.01 (−0.01, 0.03)
		180	0.29 ± 0.04	0.29 ± 0.03	0.93 (0.79, 0.98)	0.92 (0.78, 0.97)	0.00 (−0.03, 0.03)	2.72 (1.79, 3.65)	0.01 (0.01, 0.02)	0.01 (−0.01, 0.03)
		240	0.30 ± 0.03	0.30 ± 0.03	0.92 (0.78, 0.98)	0.92 (0.78, 0.97)	0.01 (−0.02, 0.03)	2.55 (1.54, 3.55)	0.01 (0.01, 0.01)	0.01 (−0.01, 0.03)
		300	0.29 ± 0.04	0.29 ± 0.04	0.96 (0.87, 0.99)	0.96 (0.88, 0.99)	0.00 (−0.02, 0.02)	2.45 (1.71, 3.18)	0.01 (0.01, 0.01)	0.01 (−0.01, 0.03)

Study 1, Houlton et al. [17]; Study 2, Houlton et al. [18]; Study 3, [19]; R1, repetition 1, R2, repetition 2; RFD, rate of force development.

**Table 5:** Performance expression stability (PES) statistics for total complex rest period (TCRP) and squat jump protocols. Between-participant PES is estimated using Pearson’s correlation coefficient (*r*) with 95% confidence interval (95% CI) and intra-class correlation coefficient (ICC_3,1_) with 95% CI. Within-participant PES is estimated using relative mean bias (RMB) with 95% limit of agreement (95% LoA), coefficient of variation with 95% CI, standard error of measure (SEM) with 95% CI and smallest worthwhile change (SWC) with 95% CI.

Variable	Study	TCRP (s)	R1	R2	Between Participant PES		Within Participant PES			
					*r* (95% CI)	ICC_3,1_ (95% CI)	RMB (95% LoA)	CV (95% CI)	SEM	SWC (95% CI)
Propulsive Impulse (N∙s)	1	0	235.53 ± 54.43	233.37 ± 56.22	0.86 (0.61, 0.96)	0.87 (0.64, 0.96)	2.16 (−54.89, 59.22)	4.99 (1.38, 8.60)	20.58 (14.92, 33.16)	19.01 (−9.44, 47.46)
		60	218.52 ± 46.48	221.81 ± 43.77	0.92 (0.77, 0.98)	0.92 (0.78, 0.98)	−3.29 (−38.46, 31.87)	4.08 (1.64, 6.53)	12.69 (9.20, 20.44)	15.52 (−7.71, 38.74)
		120	217.65 ± 48.16	212.44 ± 43.40	0.97 (0.90, 0.99)	0.96 (0.89, 0.99)	5.21 (−19.42, 29.83)	3.22 (1.81, 4.63)	8.88 (6.44, 14.31)	15.77 (−7.83, 39.38)
		180	221.45 ± 41.50	219.99 ± 41.61	0.94 (0.82, 0.98)	0.94 (0.83, 0.98)	1.46 (−26.80, 29.72)	3.60 (1.80, 5.40)	10.19 (7.39, 16.42)	14.27 (−7.09, 35.64)
		240	221.23 ± 38.42	216.81 ± 36.79	0.88 (0.65, 0.96)	0.88 (0.67, 0.96)	4.42 (−32.42, 40.98)	4.73 (2.53, 6.93)	13.19 (9.56, 21.25)	12.94 (−6.43, 32.32)
		300	217.39 ± 37.72	216.27 ± 37.09	0.87 (0.63, 0.96)	0.88 (0.66, 0.96)	1.12 (−36.38, 38.63)	5.19 (3.10, 7.27)	13.53 (9.81, 21.80)	12.85 (−6.38, 32.08)
	2	60	218.96 ± 28.26	221.24 ± 26.90	0.96 (0.89, 0.99)	0.96 (0.89, 0.99)	−2.51 (−18.30, 13.29)	2.12 (1.28, 2.96)	5.70 (4.24, 8.67)	9.75 (−4.84, 24.35)
		120	218.70 ± 29.21	222.61 ± 32.46	0.98 (0.94, 0.99)	0.98 (0.94, 0.99)	−1.89 (−15.26, 11.49)	1.83 (1.34, 2.32)	4.82 (3.59, 7.34)	11.19 (−5.56, 27.95)
		180	223.84 ± 31.68	220.80 ± 30.67	0.97 (0.92, 0.99)	0.97 (0.92, 0.99)	1.56 (−12.71, 15.83)	1.94 (1.34, 2.54)	5.15 (3.83, 7.84)	10.52 (−5.23, 26.27)
		240	219.12 ± 30.79	225.97 ± 27.06	0.87 (0.68, 0.95)	0.86 (0.65, 0.95)	−3.97 (−34.70, 26.76)	3.35 (1.12, 5.58)	11.09 (8.26, 16.87)	10.13 (−5.03, 25.29)
		300	222.19 ± 30.29	221.18 ± 29.81	0.94 (0.83, 0.98)	0.94 (0.85, 0.98)	0.67 (−19.72, 21.05)	2.80 (2.25, 3.35)	7.36 (5.48, 11.19)	10.14 (−5.04, 25.31)
	3	60	227.71 ± 28.24	227.09 ± 26.33	0.93 (0.79, 0.98)	0.93 (0.80, 0.98)	0.53 (−19.58, 20.64)	2.53 (1.65, 3.41)	7.26 (5.31, 11.44)	9.05 (−4.50, 22.60)
		120	232.11 ± 28.61	230.44 ± 25.59	0.92 (0.76, 0.97)	0.91 (0.76, 0.97)	0.40 (−22.42, 23.22)	2.55 (1.37, 3.73)	8.23 (6.03, 12.99)	9.25 (−4.59, 23.10)
		180	228.27 ± 29.66	228.43 ± 28.68	0.97 (0.90, 0.99)	0.97 (0.91, 0.99)	0.46 (−13.99, 14.90)	1.80 (1.17, 2.44)	5.21 (3.81, 8.22)	9.72 (−4.83, 24.26)
		240	232.05 ± 21.78	232.12 ± 25.68	0.81 (0.51, 0.94)	0.81 (0.53, 0.93)	2.52 (−26.32, 31.35)	3.00 (1.88, 4.12)	10.40 (7.62, 16.41)	8.13 (−4.04, 20.29)
		300	235.59 ± 27.43	232.82 ± 23.51	0.95 (0.85, 0.98)	0.94 (0.82, 0.98)	2.57 (−15.55, 20.68)	2.06 (0.81, 3.31)	6.54 (4.79, 10.31)	8.97 (−4.45, 22.39)
Peak Force (N)	1	0	1875.90 ± 275.43	1844.88 ± 232.70	0.99 (0.96, 0.99)	0.97 (0.92, 0.99)	31.03 (−84.43, 146.48)	1.56 (0.78, 2.33)	41.65 (30.20, 67.10)	87.74 (−43.58, 219.06)
		60	1923.71 ± 296.85	1907.83 ± 281.99	0.97 (0.90, 0.99)	0.97 (0.90, 0.99)	15.87 (−129.13, 160.89)	2.00 (1.09, 2.91)	52.32 (37.93, 84.28)	99.48 (−49.40, 248.36)
		120	1908.65 ± 289.61	1880.62 ± 258.83	0.98 (0.94, 0.99)	0.98 (0.92, 0.99)	28.02 (−92.24, 148.29)	1.18 (0.34, 2.03)	43.39 (31.45, 69.90)	94.46 (−46.91, 235.84)
		180	1946.94 ± 345.53	1938.42 ± 337.89	0.96 (0.89, 0.99)	0.97 (0.90, 0.99)	8.52 (−171.60, 189.00)	2.19 (0.96, 3.42)	65.11 (47.20, 104.90)	117.38 (−58.30, 293.06)
		240	1924.61 ± 298.31	1896.40 ± 259.47	0.96 (0.88, 0.99)	0.95 (0.86, 0.99)	28.21 (−139.03, 195.44)	2.01 (1.01, 3.02)	60.33 (43.74, 97.20)	96.15 (−47.75, 240.05)
		300	1953.09 ± 364.50	1940.33 ± 348.66	0.95 (0.85, 0.98)	0.95 (0.86, 0.98)	12.76 (−210.62, 236.14)	2.73 (1.35, 4.11)	80.59 (58.42, 129.83)	122.52 (−60.85, 305.89)
	2	60	1922.72 ± 273.46	1902.03 ± 315.79	0.96 (0.89, 0.99)	0.96 (0.88, 0.98)	5.91 (−157.66, 169.48)	2.12 (1.19, 3.05)	59.01 (43.95, 89.81)	94.36 (−46.86, 235.58)
		120	1895.69 ± 260.25	1881.26 ± 293.15	0.96 (0.88, 0.99)	0.95 (0.88, 0.98)	1.48 (−157.11, 160.06)	2.44 (1.61, 3.26)	57.21 (42.61, 87.07)	88.26 (−43.83, 220.35)
		180	1894.02 ± 260.61	1931.50 ± 309.61	0.97 (0.91, 0.99)	0.95 (0.88, 0.98)	−30.20 (−189.12, 128.72)	2.65 (1.82, 3.47)	57.33 (42.70, 87.26)	92.09 (−45.74, 229.92)
		240	1918.21 ± 284.87	1912.45 ± 269.66	0.98 (0.94, 0.99)	0.98 (0.94, 0.99)	5.03 (−109.33, 119.38)	1.59 (0.89, 2.30)	41.25 (30.73, 62.79)	90.95 (−45.17, 227.06)
		300	1899.10 ± 280.91	1901.84 ± 288.32	0.96 (0.90, 0.99)	0.97 (0.91, 0.99)	7.61 (−130.56, 145.77)	2.12 (1.55, 2.68)	49.85 (37.12, 75.86)	90.62 (−45.01, 226.25)
	3	60	1940.95 ± 278.38	1936.16 ± 220.03	0.94 (0.83, 0.98)	0.93 (0.79, 0.97)	6.02 (−186.50, 198.54)	2.53 (0.83, 4.23)	69.45 (50.85, 109.54)	84.85 (−42.14, 211.84)
		120	1920.86 ± 280.18	1948.57 ± 315.24	0.96 (0.89, 0.99)	0.96 (0.87, 0.99)	−21.99 (−190.91, 146.93)	2.11 (1.07, 3.15)	60.94 (44.62, 96.11)	99.30 (−49.32, 247.93)
		180	1934.26 ± 253.65	1912.07 ± 270.83	0.96 (0.88, 0.99)	0.96 (0.87, 0.99)	25.47 (−122.71, 173.65)	2.58 (1.88, 3.28)	53.46 (39.14, 84.31)	87.58 (−43.50, 218.66)
		240	1907.49 ± 225.59	1886.79 ± 216.02	0.92 (0.77, 0.97)	0.92 (0.78, 0.97)	20.84 (−146.56, 188.25)	2.16 (0.89, 3.43)	60.39 (44.22, 95.25)	74.24 (−36.87, 185.35)
		300	1914.56 ± 278.21	1878.70 ± 255.67	0.97 (0.92, 0.99)	0.97 (0.92, 0.99)	34.14 (−88.91, 157.20)	2.13 (1.48, 2.79)	44.39 (32.50, 70.01)	89.42 (−44.41, 223.24)
Mean Force (N)	1	0	1387.06 ± 145.98	1358.61 ± 131.63	0.96 (0.86, 0.99)	0.95 (0.85, 0.98)	28.44 (−57.32, 114.21)	1.59 (0.57, 2.62)	30.94 (22.43, 49.85)	48.00 (−23.84, 119.85)
		60	1425.30 ± 191.46	1405.98 ± 167.52	0.96 (0.87, 0.99)	0.95 (0.85, 0.98)	19.32 (−92.07, 130.71)	1.88 (0.94, 2.83)	40.19 (29.13, 64.74)	61.88 (−30.73, 154.49)
		120	1382.65 ± 151.76	1369.17 ± 154.11	0.92 (0.77, 0.98)	0.93 (0.79, 0.98)	13.74 (−103.37, 130.32)	2.08 (1.04, 3.12)	42.15 (30.56, 67.91)	52.58 (−26.11, 131.28)
		180	1427.63 ± 229.42	1408.53 ± 199.79	0.99 (0.92, 0.99)	0.98 (0.93, 0.99)	19.09 (−71.97, 110.16)	1.59 (0.87, 2.30)	32.85 (23.82, 52.93)	73.96 (−36.73, 184.65)
		240	1417.99 ± 160.31	1405.56 ± 165.71	0.93 (0.80, 0.98)	0.94 (0.81, 0.98)	12.43 (−103.84, 128.70)	2.14 (1.16, 3.12)	41.95 (30.41, 67.58)	56.04 (−27.83, 139.91)
		300	1442.47 ± 217.40	1405.20 ± 187.29	0.97 (0.90, 0.99)	0.96 (0.87, 0.99)	37.27 (−79.03, 153.58)	2.36 (1.24, 3.48)	41.96 (30.42, 67.60)	70.01 (−34.77, 174.78)
	2	60	1395.14 ± 132.39	1385.70 ± 134.52	0.96 (0.89, 0.99)	0.96 (0.89, 0.99)	14.25 (−56.05, 84.55)	1.66 (1.13, 2.20)	25.36 (18.89, 38.60)	43.21 (−21.46, 107.88)
		120	1386.57 ± 117.57	1388.02 ± 135.68	0.98 (0.95, 0.99)	0.98 (0.93, 0.99)	−3.47 (−65.41, 58.47)	1.23 (0.72, 1.74)	22.35 (16.64, 34.01)	47.98 (−23.83, 119.79)
		180	1378.60 ± 133.71	1372.55 ± 126.85	0.94 (0.83, 0.98)	0.94 (0.85, 0.98)	4.51 (−85.31, 95.32)	2.02 (1.49, 2.56)	32.40 (24.13, 49.32)	44.90 (−22.30, 112.10)
		240	1385.60 ± 96.62	1383.51 ± 98.75	0.97 (0.91, 0.99)	0.97 (0.92, 0.99)	−0.23 (−65.29, 64.84)	1.22 (0.81, 1.63)	23.47 (17.48, 35.72)	44.11 (−21.91, 110.13)
		300	1405.98 ± 124.27	1383.73 ± 123.78	0.95 (0.86, 0.99)	0.95 (0.86, 0.99)	30.50 (−47.50, 108.50)	1.94 (1.41, 2.47)	28.14 (20.96, 42.83)	42.11 (−20.91, 105.14)
	3	60	1393.56 ± 145.91	1376.07 ± 133.71	0.91 (0.74, 0.97)	0.91 (0.74, 0.97)	8.07 (−114.68, 130.83)	2.23 (1.26, 3.19)	44.29 (32.42, 69.85)	48.19 (−23.93, 120.31)
		120	1375.16 ± 112.97	1376.63 ± 125.99	0.91 (0.73, 0.97)	0.91 (0.74, 0.97)	1.85 (−100.88, 104.58)	2.01 (1.08, 2.95)	37.06 (27.13, 58.45)	40.02 (−19.87, 99.91)
		180	1379.88 ± 122.27	1381.75 ± 122.53	0.92 (0.78, 0.98)	0.93 (0.88, 0.98)	−1.99 (−93.91, 89.93)	1.71 (0.95, 2.47)	33.16 (24.28, 52.30)	40.82 (−20.27, 101.91)
		240	1400.49 ± 96.37	1379.22 ± 59.76	0.80 (0.47, 0.93)	0.80 (0.49, 0.93)	15.10 (−70.65, 100.76)	1.82 (1.00, 2.63)	30.92 (22.64, 48.77)	27.91 (−13.86, 69.67)
		300	1371.06 ± 118.96	1360.50 ± 104.40	0.89 (0.69, 0.96)	0.88 (0.69, 0.96)	9.01 (−99.14, 117.15)	2.12 (1.23, 3.01)	39.02 (28.56, 61.53)	38.74 (−19.24, 96.73)
Average RFD (N∙s^−1^)	1	0	4114.85 ± 1653.07	3963.88 ± 1674.71	0.87 (0.63, 0.96)	0.88 (0.66, 0.96)	150.97 (−1505.02, 1806.95)	7.61 (3.31, 11.90)	597.43 (433.11, 962.48)	572.12 (−284.14, 1428.38)
		60	4483.35 ± 1788.34	4228.94 ± 1829.55	0.60 (0.10, 0.86)	0.61 (0.15, 0.86)	254.41 (−2923.05, 3431.87)	11.74 (3.92, 19.56)	1146.30 (831.01, 1846.74)	622.99 (−309.40, 1555.37)
		120	4189.55 ± 1591.02	4087.80 ± 1710.46	0.96 (0.87, 0.99)	0.96 (0.88, 0.99)	101.75 (−847.00, 1050.50)	5.53 (2.42, 8.64)	342.28 (248.14, 551.43)	567.62 (−281.90, 1417.14)
		180	4374.64 ± 2123.74	4590.86 ± 2267.57	0.86 (0.60, 0.95)	0.86 (0.62, 0.95)	−216.23 (−2523.96, 2091.50)	9.94 (4.92, 14.96)	832.56 (603.57, 1341.28)	755.50 (−375.21, 1886.21)
		240	4483.64 ± 1697.71	4169.24 ± 1609.82	0.71 (0.29, 0.90)	0.71 (0.31, 0.90)	314.40 (−2158.55, 2787.35)	8.46 (1.65, 15.26)	892.16 (646.78, 1437.31)	570.95 (−283.56, 1425.46)
		300	4919.72 ± 2422.94	4813.58 ± 2214.17	0.89 (0.68, 0.96)	0.89 (0.70, 0.96)	106.15 (−2075.71, 2289.00)	10.82 (6.16, 15.48)	787.51 (570.91, 1268.71)	797.36 (−395.99, 1990.71)
	2	60	4249.16 ± 1911.94	4199.81 ± 2101.18	0.94 (0.83, 0.98)	0.94 (0.84, 0.98)	7.60 (−1323.55, 1337.75)	6.26 (2.63, 9.89)	479.88 (357.40, 730.34)	638.82 (−317.26, 1594.91)
		120	3941.44 ± 14,06.15	3985.81 ± 1683.08	0.97 (0.92, 0.99)	0.96 (0.89, 0.98)	−83.75 (−904.19, 736.69)	5.70 (3.64, 7.76)	295.99 (220.44, 450.47)	490.83 (−243.77, 1225.43)
		180	3761.33 ± 1524.59	3823.83 ± 1390.07	0.95 (0.85, 0.98)	0.94 (0.85, 0.98)	9.47 (−901.42, 920.36)	8.01 (5.71, 10.32)	328.62 (244.75, 500.14)	462.97 (−229.93, 1155.87)
		240	4110.30 ± 1751.20	4130.71 ± 1666.48	0.97 (0.92, 0.99)	0.97 (0.93, 0.99)	−8.66 (−754.42, 737.10)	4.59 (2.95, 6.22)	269.05 (200.38, 409.47)	538.87 (−267.62, 1345.37)
		300	4020.41 ± 2029.94	3916.84 ± 2036.74	0.96 (0.88, 0.99)	0.96 (0.89, 0.98)	103.51 (−965.76, 1172.78)	5.53 (2.60, 8.46)	385.76 (287.30, 587.10)	638.77 (−317.23, 1594.77)
	3	60	3909.70 ± 1806.33	3937.81 ± 1862.28	0.88 (0.66, 0.96)	0.88 (0.69, 0.96)	−93.12 (−1827.43, 1641.19)	8.20 (3.54, 12.87)	625.69 (458.08, 986.77)	614.91 (−305.39, 1535.21)
		120	3865.61 ± 1687.64	3755.30 ± 1777.62	0.83 (0.55, 0.94)	0.83 (0.57, 0.94)	132.37 (−1802.45, 2067.19)	9.36 (3.47, 15.25)	698.02 (511.04, 1100.85)	575.19 (−285.66, 1436.03)
		180	3835.98 ± 1462.82	3703.75 ± 1389.75	0.96 (0.87, 0.99)	0.96 (0.87, 0.99)	146.70 (−656.41, 949.81)	7.39 (5.42, 9.37)	289.74 (212.12, 456.95)	473.72 (−235.27, 1182.72)
		240	3771.87 ± 1195.12	3519.34 ± 912.87	0.80 (0.48, 0.93)	0.77 (0.44, 0.92)	268.52 (−1097.69, 1634.73)	8.99 (4.66, 13.32)	492.89 (360.86, 777.33)	357.59 (−177.59, 892.77)
		300	3718.51 ± 1464.67	3784.23 ± 1668.63	0.66 (0.23, 0.88)	0.67 (0.27, 0.88)	−58.20 (−2526.98, 2410.58)	10.33 (3.19, 17.47)	890.66 (652.07, 1404.66)	521.93 (−259.21, 1303.06)
RFD 50 ms (N∙s^−1^)	1	0	3895.13 ± 1692.45	3801.90 ± 2483.09	0.88 (0.66, 0.96)	0.83 (0.56, 0.94)	93.23 (−2391.26, 2577.71)	18.19 (12.35, 24.03)	896.32 (649.79, 1444.02)	729.99 (−362.54, 1822.52)
		60	4824.90 ± 2213.46	4784.97 ± 2639.99	0.83 (0.53, 0.94)	0.83 (0.54, 0.94)	39.93 (−2874.97,2954.82)	17.03 (11.47, 22.59)	1051.67 (762.41, 1694.28)	836.72 (−415.54, 2088.99)
		120	4188.69 ± 2505.98	4174.54 ± 2398.62	0.92 (0.76, 0.98)	0.93 (0.79, 0.98)	14.14 (−1905.27, 1933.56)	14.35 (7.72, 20.97)	692.46 (502.00, 1115.59)	842.47 (−418.40, 2103.33)
		180	4463.59 ± 2567.67	4733.46 ± 2882.60	0.85 (0.59, 0.95)	0.85 (0.60, 0.95)	−269.87 (−3227.86, 2688.12)	18.28 (12.23, 24.33)	1067.24 (773.70, 1719.37)	938.76 (−466.22, 2343.75)
		240	4971.31 ± 2786.06	4551.51 ± 2525.01	0.93 (0.79, 0.98)	0.93 (0.78, 0.98)	419.80 (−1601.97, 2441.57)	12.88 (7.44, 18.32)	729.39 (528.78, 1175.08)	916.22 (−455.03, 2287.47)
		300	5221.33 ± 2593.15	5065.96 ± 2077.50	0.87 (0.63, 0.96)	0.86 (0.61, 0.95)	155.37 (−2377.69, 2688.43)	18.33 (13.47, 23.20)	913.85 (662.50, 1472.25)	807.43 (−401.00, 2015.86)
	2	60	4384.20 ± 2607.64	4699.76 ± 2817.50	0.85 (0.57, 0.93)	0.83 (0.60, 0.94)	68.49 (−3005.45, 3142.44)	15.53 (9.89, 21.17)	1109.05 (825.99, 1687.90)	907.56 (−450.73, 2265.85)
		120	3921.31 ± 2105.98	4118.34 ± 2125.56	0.85 (0.63, 0.95)	0.85 (0.64, 0.94)	−117.04 (−3349.82, 3115.72)	18.11 (8.85, 27.37)	1166.19 (868.54, 1774.86)	1021.14 (−507.14, 2549.43)
		180	3845.63 ± 1659.59	3514.89 ± 1562.25	0.85 (0.63, 0.95)	0.86 (0.64, 0.95)	200.05 (−1706.39, 2106.48)	17.33 (10.84, 23.83)	687.78 (512.24, 1046.76)	617.74 (−306.79, 1542.28)
		240	4569.89 ± 2668.51	4399.59 ± 2178.32	0.88 (0.70, 0.96)	0.89 (0.72, 0.96)	73.34 (−2807.76, 2954.44)	17.60 (14.15, 21.06)	1039.23 (773.99, 1581.63)	1043.84 (−518.41, 2606.08)
		300	4779.04 ± 2543.93	4382.11 ± 2063.08	0.85 (0.62, 0.94)	0.82 (0.56, 0.93)	644.84 (−2198.26, 3487.93)	18.50 (13.01, 24.00)	1025.67 (763.89, 1561.00)	832.50 (−413.45, 2078.45)
	3	60	4215.71 ± 2694.18	4634.31 ± 2831.57	0.92 (0.78, 0.97)	0.92 (0.78, 0.97)	−471.75 (−2634.47, 1690.98)	14.20 (8.85, 19.55)	780.24 (571.24, 1230.52)	953.32 (−473.45, 2380.10)
		120	4001.69 ± 2232.61	4106.21 ± 2190.70	0.92 (0.78, 0.98)	0.93 (0.80, 0.98)	−112.12 (−1769.42, 1545.18)	14.63 (9.70, 19.56)	597.90 (437.74, 942.95)	744.06 −369.53, 1857.65)
		180	4291.47 ± 1859.96	4046.46 ± 2003.47	0.85 (0.60, 0.95)	0.85 (0.62, 0.95)	216.40 (−1880.05, 2312.85)	13.20 (5.44, 20.96)	756.33 (553.73, 1192.81)	669.83 (−332.66, 1672.33)
		240	4783.99 ± 2155.44	4401.64 ± 1697.24	0.79 (0.47, 0.93)	0.77 (0.43, 0.92)	431.49 (−2123.30, 2986.28)	18.62 (13.63, 23.61)	921.69 (674.79, 1453.59)	659.16 (−327.36, 1645.69)
		300	4248.63 ± 1936.19	4348.40 ± 2367.66	0.76 (0.41, 0.92)	0.77 (0.44, 0.92)	46.67 (−2966.87, 3060.20)	19.56 (14.97, 24.15)	1087.20 (795.97, 1714.62)	758.38 (−376.64, 1893.41)
RFD 100 ms (N∙s^−1^)	1	0	5354.12 ± 1568.64	4948.33 ± 2380.98	0.76 (0.39, 0.92)	0.70 (0.30, 0.89)	405.79 (−2646.53, 3458.12)	18.53 (6.67, 30.39)	1101.36 (798.44, 1774.34)	696.23 (−345.77, 1738.23)
		60	5918.82 ± 1731.87	5816.50 ± 2215.72	0.57 (0.06, 0.85)	0.57 (0.09, 0.84)	102.32 (−3583.09, 3787.74)	13.33 (5.85, 20.80)	1329.66 (963.94, 2142.14)	683.23 (−339.32, 1705.78)
		120	5132.54 ± 1415.19	4862.91 ± 1543.41	0.79 (0.44, 0.93)	0.78 (0.45, 0.93)	269.62 (−1645.42, 2184.66)	11.56 (6.57, 16.55)	690.89 (500.86, 1113.05)	510.82 (−253.69, 1275.33)
		180	5435.71 ± 1385.03	5442.48 ± 1648.05	0.61 (0.12, 0.86)	0.62 (0.16, 0.86)	−6.76 (−2671.92, 2658.39)	14.56 (9.56, 19.55)	961.51 (697.05, 1549.03)	522.82 (−259.65, 1305.30)
		240	6078.91 ± 2157.26	5974.54 ± 2042.67	0.85 (0.59, 0.95)	0.86 (0.62, 0.95)	104.37 (−2129.01, 2337.76)	12.24 (7.43, 17.06)	805.74 (584.12, 1298.07)	721.76 (−358.45, 1801.96)
		300	6389.06 ± 2453.68	6129.89 ± 2523.35	0.73 (0.33, 0.91)	0.74 (0.37, 0.91)	259.16 (−3322.55, 3840.88)	17.97 (12.49, 23.44)	1292.28 (936.85, 2081.93)	856.03 (−425.13, 2137.19)
	2	60	5082.39 ± 2066.45	4804.21 ± 1887.14	0.89 (0.73, 0.96)	0.88 (0.71, 0.96)	484.41 (−1324.92, 2293.75)	12.89 (6.18, 19.59)	652.75 (486.15, 993.44)	661.09 (−328.32, 1650.50)
		120	4527.71 ± 1442.75	4954.64 ± 1838.54	0.89 (0.73, 0.96)	0.89 (0.71, 0.96)	−295.72 (−2105.31, 1513.86)	9.46 (4.75, 14.17)	652.84 (486.22, 993.58)	665.59 (−330.55, 1661.73)
		180	4841.77 ± 1599.45	4469.51 ± 1445.68	0.80 (0.52, 0.93)	0.80 (0.53, 0.92)	258.37 (−1837.90, 2354.63)	10.01 (4.95, 15.06)	756.27 (563.24, 1150.98)	586.50 (−291.27, 1464.27)
		240	4783.89 ± 1393.94	4796.86 ± 1764.43	0.82 (0.57, 0.93)	0.82 (0.57, 0.93)	20.14 (−2346.14, 2386.42)	12.98 (6.74, 19.21)	853.68 (635.79, 1299.24)	670.75 (−333.12, 1674.61)
		300	5625.12 ± 2311.17	5206.58 ± 2372.63	0.91 (0.75, 0.97)	0.91 (0.76, 0.97)	460.31 (−1602.11, 2522.73)	13.72 (8.82, 18.62)	744.06 (554.15, 1132.40)	841.20 (−417.77, 2100.18)
	3	60	4669.23 ± 2362.88	4726.81 ± 2219.64	0.80 (0.49, 0.93)	0.81 (0.53, 0.93)	−178.27 (−2928.23, 2571.70)	16.70 (7.82, 25.57)	992.10 (726.34, 1564.64)	767.78 (−381.31, 1916.86)
		120	4647.05 ± 1787.45	4433.12 ± 1901.96	0.85 (0.60, 0.95)	0.85 (0.61, 0.95)	254.53 (−1673.23, 2182.29)	14.63 (9.35, 19.91)	695.47 (509.18, 1096.83)	619.07 (−307.45, 1545.60)
		180	4639.81 ± 1776.56	4610.33 ± 1603.60	0.88 (0.67, 0.96)	0.89 (0.70, 0.96)	−0.35 (−1597.10, 1596.41)	11.08 (6.83, 15.32)	576.06 (421.75, 908.50)	567.71 (−281.95, 1417.37)
		240	5117.61 ± 1708.01	4626.65 ± 1344.14	0.76 (0.41, 0.92)	0.74 (0.38, 0.90)	564.23 (−1573.74, 2702.20)	12.99 (7.54, 18.44)	771.31 (564.70, 1216.44)	525.00 (−260.73, 1310.74)
		300	4738.38 ± 1904.98	4586.94 ± 1792.75	0.67 (0.24, 0.88)	0.68 (0.28, 0.88)	201.56 (−2659.55, 3062.67)	17.92 (10.93, 24.91)	1031.99( 755.55, 1627.55)	615.27 (−305.57, 1536.12)
RFD 150 ms (N∙s^−1^)	1	0	5012.85 ± 1348.87	4775.19 ± 1219.69	0.79 (0.45, 0.93)	0.79 (0.46, 0.93)	237.66 (−1410.75, 1886.07)	10.64 (6.39, 14.89)	594.69 (431.13, 958.08)	443.68 (−220.35, 1107.70)
		60	5060.57 ± 1111.31	4792.86 ± 1235.19	0.55 (0.02, 0.83)	0.55 (0.05, 0.83)	267.71 (−1936.38, 2471.79)	12.11 (7.71, 16.50)	795.16 (576.46, 1281.04)	579.94 (−288.02, 1447.91)
		120	5262.01 ± 1504.61	4937.95 ± 1661.35	0.89 (0.67, 0.96)	0.88 (0.67, 0.96)	324.06 (−1176.69, 1834.81)	11.34 (9.32, 13.36)	545.03 (395.12, 878.07)	547.41 (−271.86, 1366.68)
		180	5279.00 ± 2166.13	5244.30 ± 1837.21	0.81 (0.48, 0.94)	0.81 (0.50, 0.93)	34.71 (−2498.47, 2567.89)	10.04 (5.44, 14.63)	913.89 (662.53, 1472.32)	689.83 (−342.60, 1722.26)
		240	4946.02 ± 1430.07	4776.96 ± 1553.72	0.57 (0.06, 0.85)	0.59 (0.11, 0.85)	169.07 (−2545.89, 2884.02)	15.89 (10.17, 21.62)	979.47 (710.07, 1577.97)	513.73 (−255.13, 1282.59)
		300	5888.25 ± 2397.93	5848.89 ± 2049.63	0.74 (0.35, 0.91)	0.75 (0.39, 0.91)	39.36 (−3143.14, 3221.86)	14.18 (9.35, 19.00)	1148.04 (832.28, 1849.54)	766.14 (−380.49, 1912.78)
	2	60	4654.40 ± 1796.15	4500.69 ± 1438.56	0.74 (0.57, 0.94)	0.81 (0.55, 0.93)	214.41 (−1611.79, 2040.61)	13.68 (8.14, 19.22)	658.84 (490.68, 1002.70)	518.19 (−257.35, 1293.73)
		120	4238.92 ± 1151.67	4197.38 ± 1424.42	0.79 (0.49, 0.92)	0.77 (0.47, 0.91)	139.61 (−1711.97, 1991.19)	13.34 (9.30, 17.38)	667.99 (497.50, 1016.64)	470.95 (−233.89, 1175.79)
		180	4254.64 ± 1698.21	4450.76 ± 1090.22	0.75 (0.42, 0.90)	0.71 (0.36, 0.88)	−147.09 (−2214.32, 1920.15)	15.43 (10.08, 20.77)	745.79 (555.44, 1135.05)	468.03 (−232.44, 1168.50)
		240	4245.26 ± 1362.82	4373.70 ± 1318.56	0.81 (0.54, 0.93)	0.82 (0.57, 0.93)	−125.41 (−1847.37, 1596.56)	11.45 (7.91, 14.99)	621.23 (462.67, 945.47)	492.65 (−244.67, 1229.98)
		300	4805.50 ± 1929.69	4442.78 ± 2061.56	0.81 (0.53, 0.93)	0.81 (0.54, 0.93)	360.21 (−1938.37, 2658.78)	15.17 (11.10, 19.23)	829.25 (617.60, 1262.07)	678.23 (−336.83, 1693.29)
	3	60	4174.70 ± 1906.28	4199.27 ± 1836.76	0.87 (0.63, 0.95)	0.87 (0.65, 0.95)	−164.33 (−2070.83, 1742.17)	15.43 (8.94, 21.91)	687.81 (503.56, 1084.74)	633.84 (−314.79, 1582.48)
		120	4077.52 ± 1547.31	4094.31 ± 1452.71	0.79 (0.46, 0.93)	0.80 (0.50, 0.93)	−59.92 (−1925.15, 1805.30)	13.77 (10.62, 16.93)	672.91 (492.66, 1061.25)	498.61 (−247.63, 1244.85)
		180	4025.04 ± 1746.51	3991.79 ± 1136.02	0.73 (0.35, 0.90)	0.69 (0.29, 0.88)	−1.61 (−2271.33, 2268.10)	16.71 (10.81, 22.62)	818.84 (599.50, 1291.40)	488.79 (−242.75, 1220.34)
		240	4292.82 ± 1512.57	4186.49 ± 826.52	0.75 (0.38, 0.91)	0.65 (0.22, 0.87)	150.86 (−1822.90, 2124.61)	14.32 (6.68, 21.95)	712.07 (521.33, 1123.01)	406.33 (−201.80, 1014.47)
		300	4325.66 ± 1463.12	4177.18 ± 1429.75	0.77 (0.42, 0.92)	0.78 (0.45, 0.92)	119.65 (−1811.61, 2050.90)	11.33 (7.08, 15.59)	696.74 (510.10, 1098.82)	494.92 (−245.79, 1235.63)
Propulsion Time (s)	1	0	0.37 ± 0.06	0.38 ± 0.07	0.88 (0.65, 0.96)	0.87 (0.64, 0.96)	−0.01 (−0.07, 0.05)	4.04 (1.74, 6.34)	0.02 (0.02, 0.04)	0.02 (−0.01, 0.05)
		60	0.36 ± 0.06	0.36 ± 0.07	0.89 (0.69, 0.97)	0.89 (0.69, 0.96)	−0.01 (−0.07, 0.05)	4.00 (1.61, 6.40)	0.02 (0.02, 0.03)	0.02 (−0.01, 0.06)
		120	0.37 ± 0.06	0.37 ± 0.05	0.85 (0.59, 0.95)	0.86 (0.61, 0.95)	0.00 (−0.07, 0.06)	4.24 (1.95, 6.53)	0.02 (0.02, 0.04)	0.02 (−0.01, 0.05)
		180	0.36 ± 0.07	0.36 ± 0.06	0.94 (0.80, 0.98)	0.94 (0.82, 0.98)	0.00 (−0.05, 0.04)	4.11 (2.42, 5.79)	0.02 (0.01, 0.03)	0.02 (−0.01, 0.06)
		240	0.36 ± 0.06	0.36 ± 0.05	0.90 (0.71, 0.97)	0.90 (0.73, 0.97)	0.00 (−0.05, 0.05)	3.75 (2.04, 5.46)	0.02 (0.01, 0.03)	0.02 (−0.01, 0.05)
		300	0.34 ± 0.06	0.36 ± 0.05	0.88 (0.66, 0.96)	0.88 (0.66, 0.96)	−0.01 (−0.07, 0.04)	5.06 (3.00, 7.12)	0.02 (0.01, 0.03)	0.02 (−0.01, 0.05)
	2	60	0.38 ± 0.07	0.38 ± 0.07	0.91 (0.76, 0.97)	0.91 (0.77, 0.97)	−0.01 (−0.06, 0.05)	3.54 (1.92, 5.16)	0.02 (0.01, 0.03)	0.02 (−0.01, 0.05)
		120	0.38 ± 0.05	0.38 ± 0.05	0.95 (0.85, 0.98)	0.94 (0.85, 0.98)	0.00 (−0.04, 0.03)	2.39 (1.54, 3.24)	0.01 (0.01, 0.02)	0.02 (−0.01, 0.04)
		180	0.39 ± 0.07	0.38 ± 0.05	0.91 (0.76, 0.97)	0.90 (0.74, 0.96)	0.00 (−0.05, 0.05)	3.80 (2.77, 4.83)	0.02 (0.01, 0.03)	0.02 (−0.01, 0.05)
		240	0.38 ± 0.06	0.38 ± 0.06	0.91 (0.76, 0.97)	0.97 (0.91, 0.99)	0.00 (−0.05, 0.05)	2.26 (1.60, 2.93)	0.01 (0.01, 0.02)	0.02 (−0.01, 0.05)
		300	0.37 ± 0.06	0.38 ± 0.06	0.95 (0.87, 0.98)	0.95 (0.88, 0.98)	−0.01 (−0.05, 0.03)	3.04 (1.89, 4.19)	0.01 (0.01, 0.02)	0.02 (−0.01, 0.05)
	3	60	0.39 ± 0.08	0.40 ± 0.09	0.87 (0.64, 0.96)	0.87 (0.66, 0.95)	0.00 (−0.08, 0.09)	4.13 (2.27, 6.00)	0.03 (0.02, 0.05)	0.03 (−0.01, 0.07)
		120	0.39 ± 0.06	0.40 ± 0.07	0.88 (0.67, 0.96)	0.88 (0.67, 0.96)	−0.01 (−0.07, 0.05)	3.71 (1.54, 5.87)	0.02 (0.02, 0.03)	0.02 (−0.01, 0.05)
		180	0.39 ± 0.07	0.39 ± 0.07	0.90 (0.72, 0.97)	0.91 (0.74, 0.97)	0.00 (−0.06, 0.05)	3.60 (2.12, 5.09)	0.02 (0.01, 0.03)	0.02 (−0.01, 0.06)
		240	0.38 ± 0.05	0.39 ± 0.05	0.88 (0.68, 0.96)	0.88 (0.69, 0.96)	−0.02 (−0.06, 0.03)	4.00 (2.28, 5.72)	0.02 (0.01, 0.03)	0.02 (−0.01, 0.04)
		300	0.39 ± 0.06	0.40 ± 0.06	0.86 (0.62, 0.95)	0.86 (0.63, 0.95)	−0.01 (−0.07, 0.05)	4.19 (2.16, 6.21)	0.02 (0.02, 0.04)	0.02 (−0.01, 0.05)

Study 1, Houlton et al. [17]; Study 2, Houlton et al. [18]; Study 3, [19]; R1, repetition 1, R2, repetition 2; RFD, rate of force development.

## Data Availability

The raw data supporting the conclusions of this article will be made available by the authors upon request.

## Abbreviations

The following abbreviations are used in this manuscript:

3RM	Three-repetition maximum
ANOVA	Analysis of variance
CCT	Complex–contrast training
CI	Confidence intervals
CMJ	Countermovement jump
CV	Coefficient of variation
ICC	Intraclass correlation coefficient
ICRP	Intra-contrast rest period
J_PROP_	Propulsive impulse
LoA	Limits of agreement
MF	Mean force
PAPE	Post-activation performance enhancement
PES	Performance expression stability
PF	Peak force
RFD	Rate of force development
RMB	Relative mean bias
RR	Rest redistribution
SEM	Standard error of measurement
SJ	Squat jump
SWC	Smallest worthwhile change
TCRP	Total contrast rest period
t_PROP_	Propulsion time
